# Theoretical and numerical analysis of COVID-19 pandemic model with non-local and non-singular kernels

**DOI:** 10.1038/s41598-022-21372-4

**Published:** 2022-10-28

**Authors:** Ting Cui, Peijiang Liu, Anwarud Din, Fawad Ali

**Affiliations:** 1 School of Economics, Guangdong University of Finance and Economics, Guangzhou, 510320 People’s Republic of China; 2 School of Statistics and Mathematics, Guangdong University of Finance and Economics, Big data and Educational Statistics Application Laboratory, Guangzhou, 510320 People’s Republic of China; 3 Department of Mathematics, Sun Yat-sen University, Guangzhou, 510275 People’s Republic of China; 4 Institute of Numerical Sciences, Kohat University of Science and Technology, Kohat, KPK 26000 Pakistan

**Keywords:** Mathematics and computing, Physics

## Abstract

The global consequences of Coronavirus (COVID-19) have been evident by several hundreds of demises of human beings; hence such plagues are significantly imperative to predict. For this purpose, the mathematical formulation has been proved to be one of the best tools for the assessment of present circumstances and future predictions. In this article, we propose a fractional epidemic model of coronavirus (COVID-19) with vaccination effects. An arbitrary order model of COVID-19 is analyzed through three different fractional operators namely, Caputo, Atangana-Baleanu-Caputo (ABC), and Caputo-Fabrizio (CF), respectively. The fractional dynamics are composed of the interaction among the human population and the external environmental factors of infected peoples. It gives an extra description of the situation of the epidemic. Both the classical and modern approaches have been tested for the proposed model. The qualitative analysis has been checked through the Banach fixed point theory in the sense of a fractional operator. The stability concept of Hyers-Ulam idea is derived. The Newton interpolation scheme is applied for numerical solutions and by assigning values to different parameters. The numerical works in this research verified the analytical results. Finally, some important conclusions are drawn that might provide further basis for in-depth studies of such epidemics.

## Introduction

At this time, individuals worldwide face an epidemic brought approximately by using the SARS-CoV-2 virus. The Severe Acute Respiratory Syndrome Coronavirus 2 (SARS-CoV-2), a mineral virus present in proteins is the active source of the enormous coronavirus sickness. This is understood that it relates to a big own society of the viruses as the community of coronaviruses. Initially, excessive sickness as a result of a Coronavirus found in 2003 from acute severe respiration syndrome (SARS) epidemic taking started in China. The 2nd shift of this virus found in the middle east respiration syndrome virus (MERS) was tested in 2012 in the middle east countries of Asia UAE, Syria, Saudi Arabia, etc.^1,2^. The COVID-19 sickness of the year 2019 became the starting declaration as an Emergency by the organization of World Health (WHO) on $30^{th}$ of January 2020^3,4^ through different territories of public health. The said disease was announced as indeed a deadly infection on $11^{th}$ of March 2020^1^. The cause of this declaration is due to the determination to the public civilian health emergency to about all the Nations of globe and spreading of the disease. The infection is mainly transmitted through connection with infectious respiration or lung drops from coughing, sneezing, and speeching^5–7^. More analysis has proved that the illness may be caused via air-borne spreadings^8,9^. Contact with infectious areas is also a common reason for contamination. Few tested symptoms of COVID-19 that may be seen after 2-14 days of exposure are coughing, high fever, windedness, pain in muscles, smell-less, diarrhea, jogging off the nose, and fatigue.

On the eleventh of January, 2021 there have been 908, 680, novel inflamed instances reported in the world 921, 222, cases in America, 4,254 confirmed cases in the country of Tennessee, and 133 were reported in the Republic of China^10^. A complete 11,415 cases of deaths have been reported internationally^10,11^. The continent of America recently has over 40 million verified infections with the united states main on the top in all other Nations in this vicinity and the Sector with nearly 25 million showed instances. At the same time as there appears to be a rapidly growing variety of confirmed instances, there also are many intervention applications given to reduce the cutting-edge traits of the disorder. Such packages consist of the contemporary vaccines packages which all started inside the remaining region of 2020^14^, use of dis-infections, community distancing, public fitness schooling, use of nostril masks and other covering shields, isolations of inflamed/uncovered humans, investment of COVID-19 tasks and so on.

The mathematical formulation concepts describe the real world situation very well up to small errors and these phenomena are called mathematical modeling^12,13^. This concept is applied to various biological, business problems, and different dynamics phenomena. Therefore these aspects can also be used as a study of spreading and controlling the diseases and future predictions for the sake of mankind. So various research articles have been published related to COVID-19 which can be seen in (^15–18^). Controlling and minimizing techniques for the said pandemic from further spreading, are the main and biggest challenges for the recent researchers and different scholars around the globe. Therefore some work has been done against the said disease and made some beneficial plans and strategies for its optimality and elimination for the society. The dynamical problems and the infectious disease situation are handled by mathematical modeling. Such types of techniques are very well to enable the situation of COVID-19 in the community (see^19–21^).

Modern calculus is the generalization of the integer order calculus having an extra degree of choices for analysis. To check the inside behavior of the dynamics of various problems we can use significantly the idea of fractional calculus. Fractional dynamical systems can be checked on any values lying between two different natural numbers. Therefore fractional order differential equation may model very well the infectious problems under discussions^30–33^. So many fractional operators have been defined as having a kernel of singularity and non-singularity^34–37^ along with better applicability^30,38,39^. Some of the scholars in^40^ have taken a problem related to the coupled dynamics of hepatitis and cancer under the fractional operators along with their valuable results.

The remaining article is constructed in the following format: in "Preliminaries" section includes the basic definitions of the fractional-order derivatives. The model construction processes in form of integer and fractional order derivatives are presented in "The classical integer order model" section. The existence of a solution is pointed out in "Existence results" section through fixed point theory in sense of the Atangana-Baleanu-Caputo derivative. U-H stability concept is established in "Hyers-Ulam stability" section. The graphical representation is carried out in "Numerical schemes and graphical results" section, while a short summary is added in the last section.

## Preliminaries

We present in this section, some definitions of differential and integral operators starting with Caputo fractional derivative

1 $$\begin{aligned} { }_{0}^{C} \mathbb {D}_{t}^{\Theta } \mathcal {F}(t)=\frac{1}{\Gamma (1-\Theta )} \int _{0}^{t} \frac{d}{d \Psi } \mathcal {F}(\Psi )(t-\Psi )^{-\Theta } d \Psi . \end{aligned}$$

Caputo-Fabrizio fractional derivative

2 $$\begin{aligned} { }_{0}^{C F} \mathbb {D}_{t}^{\Theta } \mathcal {F}(t)=\frac{M(\Theta )}{1-\Theta } \int _{0}^{t} \frac{d}{d \Psi } \mathcal {F}(\Psi ) \exp \left[ -\frac{\Theta }{1-\Theta }(t-\Psi )\right] d \Psi . \end{aligned}$$

Atangana-Baleanu fractional derivative

3 $$\begin{aligned} { }^{A B C} \mathbb {D}_{t}^{\Theta } \mathcal {F}(t)=\frac{A B(\Theta )}{1-\Theta } \int _{0}^{t} \frac{d}{d \Psi } \mathcal {F}(\Psi ) E_{x}\left[ -\frac{\Theta }{1-\Theta }(t-\Psi )^{\Theta }\right] d \Psi . \end{aligned}$$

## The classical integer order model

Let us have a population that is mixed with an equal contact rate for each and every population. This consideration is of the idealistic approach of various compartments through mathematical modeling for the description of the epidemic dynamical analysis. We take the whole population $\mathcal {N}(t)$ at time *t* and make their partition in some biological conditions related to each individual’s health conditions. The population includes Susceptible class $\mathcal {S}(t)$, Vaccinated class $\mathcal {V}(t)$, Exposed individuals $\mathcal {E}(t)$, Infectious symptomatic class $\mathcal {I}(t)$, Asymptomatic infectious class $\mathcal {A}(t)$, Hospitalized individuals $\mathcal {H}(t)$ and Recovery cases $\mathcal {R}(t)$.

The mathematical model of COVID-19 in the form of integer order is given in^22^, along with some assumptions therein may be followed as:

4 $$\begin{aligned} \left\{ \begin{aligned}&\dot{\mathcal {S}}=(1-p)\Pi +\eta \mathcal {R}-(\beta _s+\mu +v)\mathcal {S},\\ {}&\dot{\mathcal {V}}= p\Pi +v\mathcal {S}-(\beta _v+\mu )\mathcal {V},\\ {}&\dot{\mathcal {E}}=\beta _s \mathcal {S}+\beta _v \mathcal {V}-(\sigma +\mu )E,\\ {}&\dot{\mathcal {I}}=\sigma \psi \mathcal {E}+\lambda (1-\phi ) \mathcal {A}-(\gamma +\mu +\delta )\mathcal {I},\\ {}&\dot{\mathcal {A}}=\sigma (1-\psi )\mathcal {E}-(\lambda +\mu )\mathcal {A},\\ {}&\dot{\mathcal {H}}= \gamma (1-\kappa )\mathcal {I}-(\tau +\mu +\delta )\mathcal {H},\\ {}&\dot{\mathcal {R}}= \gamma \kappa \mathcal {I}+\lambda \phi \mathcal {A}+\tau \mathcal {H}-(\eta +\mu )\mathcal {R}, \end{aligned}\right. \end{aligned}$$

with

5 $$\begin{aligned} \mathcal {S}(0), \mathcal {V}(0), \mathcal {E}(0), \mathcal {I}(0), \mathcal {A}(0), \mathcal {H}(0), \mathcal {R}(0) \ge 0. \end{aligned}$$

### Parameter estimation

**Figure 1:**
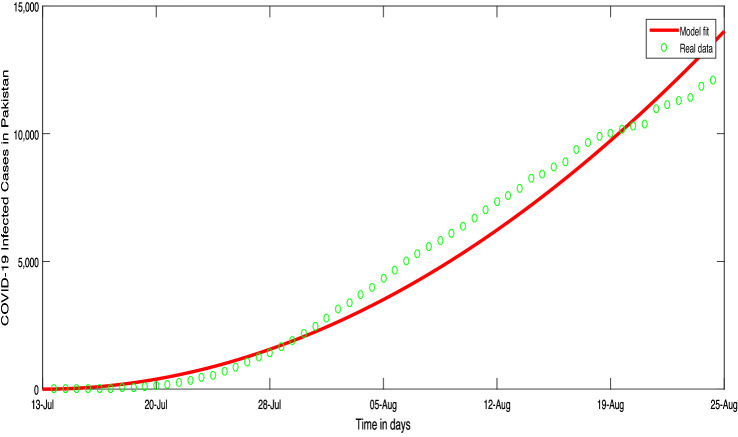
Conformed COVID-19 cumulative cases time series in Pakistan

**Figure 2:**
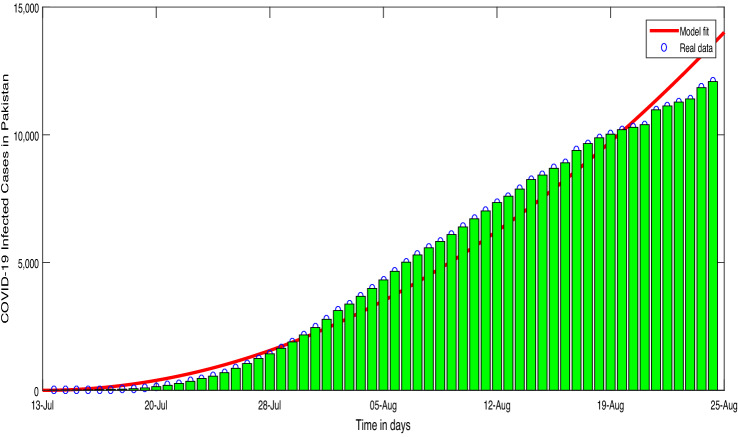
Conformed COVID-19 cumulative cases time series in Pakistan

Here we utilized the least square curve fitting for the recorded COVID-19 cases in Pakistan from 13-Jul 2021 to 25-Aug, 2021. Comparison of the model with the reported cases are shown in Figs. 1 and 2, while the estimated values of the parameters are shown in Table 1.

**Table 1:** Descriptions and numerical values of the parameters

Symbols	Description	Values	References
$\Pi$	Birth rate of population in the Susceptible class	2.3072	Fitted
*p*	Rate of proportions of new born Vaccinated population	0.0005	^22^
*v*	Rate Vaccinated peoples	0.4	^22^
$\omega _{A}$	Decrease in population through contact from $\mathcal {A}(t)$	0.076	Fitted
$\omega _{I}$	Growth in the rate of transmitted from Symptomatic class	0.1326	Fitted
$\omega _{H}$	decrease in the rate of transmitted from Hospitalized	0.6000	^22^
$\mu$	Rate of Natural occurring death	0.005	Fitted
$\delta$	The rate induction disease	0.124	Fitted
$\epsilon$	Rate of reduction trough infection of vaccination	0.00554	Fitted
$\sigma$	Rate of removal from Expose cases	0.03365	Fitted
$\gamma$	Rate of removal from the Infectious cases	0.08	^22^
$\kappa$	Rate of recovery form Infected class with out treatment	0.0941	^22^
$\psi$	Infection reduction of vaccinated individuals	0.1433	Fitted
*b*	The rate of connections	1.12	^22^
$\tau$	Rate of Recovered Hospitalized population	0.0718	^22^
$\phi$	Rate of natural recovery proportions of asymptomatic class	0.6703	Fitted
$\lambda$	Exit rate from the asymptomatic class	0.25021	Fitted
$\eta$	Losing of strong immunity rate	0.081	Fitted

### Model in Atangana-Baleanu-Caputo sense

Modeling the dynamical problems through non-integer order epidemic models was investigated in the near future by many of the field scholars^23–27^. In this subpart, we model the arbitrary-order COVID-19 dynamics. For the observation of the memory impacts, the problem (4), in the form of integration as:

6 $$\begin{aligned} \left\{ \begin{aligned}&^{\mathcal {ABC}} \mathbb {D}_{0, t}^{\Theta }[\mathcal {S}]= (1-p)\Pi +\eta \mathcal {R}-(\beta _s+\mu +v) \mathcal {S},\\ {}&^{\mathcal {ABC}} \mathbb {D}_{0, t}^{\Theta }[\mathcal {V}]= p\Pi +v \mathcal {S}-(\beta _v+\mu )\mathcal {V},\\ {}&^{\mathcal {ABC}} \mathbb {D}_{0, t}^{\Theta }[\mathcal {E}]= \beta _s \mathcal {S}+\beta _v \mathcal {V}-(\sigma +\mu )\mathcal {E},\\ {}&^{\mathcal {ABC}} \mathbb {D}_{0, t}^{\Theta }[\mathcal {I}] =\sigma \psi \mathcal {E}+\lambda (1-\phi )\mathcal {A}-(\gamma +\mu +\delta )\mathcal {I},\\ {}&^{\mathcal {ABC}} \mathbb {D}_{0, t}^{\Theta }[\mathcal {A}]=\sigma (1-\psi )\mathcal {E}-(\lambda +\mu )\mathcal {A},\\ {}&^{\mathcal {ABC}} \mathbb {D}_{0, t}^{\Theta }[\mathcal {H}] = \gamma (1-\kappa )\mathcal {I}-(\tau +\mu +\delta )\mathcal {H},\\ {}&^{\mathcal {ABC}} \mathbb {D}_{0, t}^{\Theta }[\mathcal {R}] = \gamma \kappa \mathcal {I}+\lambda \phi \mathcal {A}+\tau \mathcal {H}-(\eta +\mu )\mathcal {R}. \end{aligned}\right. \end{aligned}$$

Under the starting approximation

$$\begin{aligned} \mathcal {S}(0)=\mathcal {S}^0, \mathcal {V}(0)=\mathcal {V}^0, \mathcal {E}(0)=\mathcal {E}^0, \mathcal {I}(0)=\mathcal {I}^0, \mathcal {A}(0)=\mathcal {A}^0, \mathcal {H}(0)=\mathcal {H}^0, \mathcal {R}(0)=\mathcal {R}^0 \ge 0. \end{aligned}$$

The transition rates from susceptible and vaccinated to exposed is given by:

7 $$\begin{aligned} \begin{aligned} \beta _s =&b\frac{\omega _{\mathcal {A}} \mathcal {A}+\omega _{\mathcal {I}} \mathcal {I}+\omega _{\mathcal {H}} \mathcal {H}}{\mathcal {N}} \\ \beta _v =&b(1-\epsilon )\frac{\omega _{\mathcal {A}} \mathcal {A}+\omega _{\mathcal {I}} \mathcal {I}+\omega _{\mathcal {H}} \mathcal {H}}{\mathcal {N}}. \end{aligned}\end{aligned}$$

## Existence results

Assume that $\mathbb {B}(J)$ represent a real-valued continuous function which containing the suprimum norm space property is a banach space on $J =[0,b]$ and $P=\mathcal {B}(J) \times \mathcal {B}(J) \times \mathcal {B}(J) \times \mathcal {B}(J) \times \mathcal {B}(J) \times \mathcal {B}(J) \times \mathcal {B}(J)$ with norm $\left\| \left( \mathcal {S}, \mathcal {V}, \mathcal {E}, \mathcal {I}, \mathcal {A}, \mathcal {H}, \mathcal {R}\right) \right\| =\Vert \mathcal {S}\Vert +\Vert \mathcal {V}\Vert +\left\| \mathcal {E}\right\| +\left\| \mathcal {I}\right\| +\Vert \mathcal {A}\Vert +\left\| \mathcal {H}\right\| +\Vert \mathcal {R}\Vert$, where $\Vert \mathcal {S}\Vert =\sup _{t \in J}|\mathcal {S}(t)|,\Vert \mathcal {V}\Vert =\sup _{t \in j}|\mathcal {V}(t)|,\left\| \mathcal {E}\right\| =\sup _{t \in j}|\mathcal {E}(t)|, \left\| \mathcal {I}\right\| =\sup _{t \in j}|\mathcal {I}(t)|, \left\| \mathcal {A}\right\| =\sup _{t \in j}|\mathcal {A}(t)|, \left\| \mathcal {H}\right\| =\sup _{t \in j}|\mathcal {H}(t)|, \left\| \mathcal {R}\right\| =\sup _{t \in j}|\mathcal {R}(t)|$. Now by setting $\mathcal {ABC}$ fractional integral operator to left and right hand sides of Eq. (6), we get the following system

8 $$\begin{aligned} \left\{ \begin{aligned}&\mathcal {S}(t)-\mathcal {S}(0)= ^{\mathcal {ABC}} \mathbb {D}_{0, t}^{\Theta }[\mathcal {S}] \left\{ (1-p)\Pi +\eta \mathcal {R}-(\beta _s+\mu +v) \mathcal {S}\right\} ,\\ {}&\mathcal {V}(t)-\mathcal {V}(0)= ^{\mathcal {ABC}} \mathbb {D}_{0, t}^{\Theta }[\mathcal {V}] \left\{ p\Pi +v \mathcal {S}-(\beta _v+\mu )\mathcal {V}\right\} ,\\ {}&\mathcal {E}(t)-\mathcal {E}(0)= ^{\mathcal {ABC}} \mathbb {D}_{0, t}^{\Theta }[\mathcal {E}] \left\{ \beta _s \mathcal {S}+\beta _v \mathcal {V}-(\sigma +\mu )\mathcal {E}\right\} , \\ {}&\mathcal {I}(t)-\mathcal {I}(0)=^{\mathcal {ABC}} \mathbb {D}_{0, t}^{\Theta }[\mathcal {I}] \left\{ \sigma \psi \mathcal {E}+\lambda (1-\phi )\mathcal {A}-(\gamma +\mu +\delta )\mathcal {I}\right\} ,\\ {}&\mathcal {A}(t)-\mathcal {A}(0)=^{\mathcal {ABC}} \mathbb {D}_{0, t}^{\Theta }[\mathcal {A}] \left\{ \sigma (1-\psi )\mathcal {E}-(\lambda +\mu )\mathcal {A}\right\} , \\ {}&\mathcal {H}(t)-\mathcal {H}(0)=^{\mathcal {ABC}} \mathbb {D}_{0, t}^{\Theta }[\mathcal {H}] \left\{ \gamma (1-\kappa ) \mathcal {I}-(\tau +\mu +\delta ) \mathcal {H} \right\} \\ {}&\mathcal {R}(t)-\mathcal {R}(0)=^{\mathcal {ABC}} \mathbb {D}_{0, t}^{\Theta }[\mathcal {R}] \left\{ \gamma \kappa \mathcal {I}+\lambda \phi \mathcal {A}+\tau \mathcal {H}-(\eta +\mu )R\right\} . \end{aligned}\right. \end{aligned}$$

Applying the definition of 3, we can write

9 $$\begin{aligned} \begin{aligned} \mathcal {S}(t)-\mathcal {S}(0)=&\frac{1-\Theta }{B(\Theta )} \mathbb {M}_{1}(\Theta , t, \mathcal {S})+\frac{\Theta }{B(\Theta ) \Gamma (\Theta )} \times \int _{0}^{t}(t-\vartheta )^{\Theta -1} \mathbb {M}_{1}(\Theta , \vartheta , \mathcal {S}(\vartheta )) d \vartheta ,\\ \mathcal {V}(t)-\mathcal {V}(0)=&\frac{1-\Theta }{B(\Theta )} \mathbb {M}_{2}(\Theta , t, \mathcal {V})+\frac{\Theta }{B(\Theta ) \Gamma (\Theta )} \times \int _{0}^{t}(t-\vartheta )^{\Theta -1} \mathbb {M}_{2}(\Theta , \vartheta , \mathcal {V}(\vartheta )) d \vartheta ,\\ \mathcal {E}(t)-\mathcal {E}(0)=&\frac{1-\Theta }{B(\Theta )} \mathbb {M}_{3}(\Theta , t, \mathcal {E})+\frac{\Theta }{B(\Theta ) \Gamma (\Theta )} \times \int _{0}^{t}(t-\vartheta )^{\Theta -1} \mathbb {M}_{3}(\Theta , \vartheta , \mathcal {E}(\vartheta )) d \vartheta ,\\ \mathcal {I}(t)-\mathcal {I}(0)=&\frac{1-\Theta }{B(\Theta )} \mathbb {M}_{4}(\Theta , t, \mathcal {I})+\frac{\Theta }{B(\Theta ) \Gamma (\Theta )} \times \int _{0}^{t}(t-\vartheta )^{\Theta -1} \mathbb {M}_{4}(\Theta , \vartheta , \mathcal {I}(\vartheta )) d \vartheta ,\\ \mathcal {A}(t)-\mathcal {A}(0)=&\frac{1-\Theta }{B(\Theta )} \mathbb {M}_{5}(\Theta , t, \mathcal {A})+\frac{\Theta }{B(\Theta ) \Gamma (\Theta )} \times \int _{0}^{t}(t-\vartheta )^{\Theta -1} \mathbb {M}_{5}(\Theta , \vartheta , \mathcal {A}(\vartheta )) d \vartheta ,\\ \mathcal {H}(t)-\mathcal {H}(0)=&\frac{1-\Theta }{B(\Theta )} \mathbb {M}_{6}(\Theta , t, \mathcal {H})+\frac{\Theta }{B(\Theta ) \Gamma (\Theta )} \times \int _{0}^{t}(t-\vartheta )^{\Theta -1} \mathbb {M}_{6}(\Theta , \vartheta , \mathcal {H}(\vartheta )) d \vartheta ,\\ \mathcal {R}(t)-\mathcal {R}(0)=&\frac{1-\Theta }{B(\Theta )} \mathbb {M}_{7}(\Theta , t, \mathcal {R})+\frac{\Theta }{B(\Theta ) \Gamma (\Theta )} \times \int _{0}^{t}(t-\vartheta )^{\Theta -1} \mathbb {M}_{7}(\Theta , \vartheta , \mathcal {R}(\vartheta )) d \vartheta . \end{aligned} \end{aligned}$$

where

10 $$\begin{aligned}&\mathbb {M}_{1}(\Theta , t, \mathcal {S}(t))= (1-p)\Pi +\eta \mathcal {R}-(\beta _s+\mu +v)\mathcal {S},\nonumber \\&\mathbb {M}_{2}(\Theta , t, \mathcal {V}(t))= p\Pi +vS-(\beta _v+\mu ) \mathcal {V},\nonumber \\&\mathbb {M}_{3}\left( \Theta , t, \mathcal {E}(t)\right) = \beta _s \mathcal {S}+\beta _v \mathcal {V}-(\sigma +\mu )\mathcal {E},\nonumber \\&\mathbb {M}_{4}\left( \Theta , t, \mathcal {I}(t)\right) = \sigma \psi \mathcal {E}+\lambda (1-\phi )\mathcal {A}-(\gamma +\mu +\delta )\mathcal {I},\nonumber \\&\mathbb {M}_{5}\left( \Theta , t, \mathcal {A}(t)\right) = \sigma (1-\psi )\mathcal {E}-(\lambda +\mu )\mathcal {A},\nonumber \\&\mathbb {M}_{6}(\Theta , t, \mathcal {H}(t))= \gamma (1-\kappa )\mathcal {I}-(\tau +\mu +\delta )\mathcal {H},\nonumber \\&\mathbb {M}_{7}(\Theta , t, \mathcal {R}(t))= \gamma \kappa I+\lambda \phi \mathcal {A}+\tau \mathcal {H}-(\eta +\mu )\mathcal {R}. \end{aligned}$$

If $\mathcal {S}, \mathcal {V}, \mathcal {E}, \mathcal {I}, \mathcal {A}, \mathcal {H}$ and $\mathcal {R}$ contains their upper greatest value or bound, then $\mathbb {M}_1$, $\mathbb {M}_{2}$, $\mathbb {M}_{3}$, $\mathbb {M}_{4}$, $\mathbb {M}_{5}$, $\mathbb {M}_{6}$ and $\mathbb {M}_{7}$ must fulfill the Lipschitz condition. Assuming that $\mathcal {S}$ and $S^*$ are two different functions, we obtain

11 $$\begin{aligned} \begin{aligned} \left\| \mathbb {M}_{1}(\lambda , t, \mathcal {S})-\mathbb {M}_{1}\left( \lambda , t, \mathcal {S}^{*}\right) \right\|&=\left\| -\left( -(\beta _s+\mu +v) \right) \left( \mathcal {S}-\mathcal {S}^{*}\right) \right\| \\&\le \left\| -\left( -(\beta _s+\mu +v) \right) \right\| \left\| \left( \mathcal {S}-\mathcal {S}^{*}\right) \right\| \\&\le (\left\| \beta _s+\mu +v\right\| ) \left\| \left( \mathcal {S}-\mathcal {S}^{*}\right) \right\| . \end{aligned} \end{aligned}$$

Taking into account

$$\begin{aligned} \eta _{1}:= \left( \beta _s+\mu +v\right) , \end{aligned}$$

one reaches

12 $$\begin{aligned} \left\| \mathbb {M}_{1}(\Theta , t, \mathcal {S})-\mathbb {M}_{1}\left( \Theta , t, \mathcal {S}^{*}\right) \right\| \le \eta _{1}\left\| \mathcal {S}-\mathcal {S}^{*}\right\| . \end{aligned}$$

In a similar way, we can get the following

13 $$\begin{aligned} \begin{array}{l} \left\| \mathbb {M}_{2}(\Theta , t, \mathcal {V})-\mathbb {M}_{2}\left( \Theta , t, \mathcal {V}^{*}\right) \right\| \le \eta _{2}\left\| \mathcal {V}-\mathcal {V}^{*}\right\| , \\ \left\| \mathbb {M}_{3}\left( \Theta , t,\mathcal {E}\right) -\mathbb {M}_{3}\left( \Theta , t, \mathcal {E}^{*}\right) \right\| \le \eta _{3}\left\| \mathcal {E}-\mathcal {E}^{*}\right\| , \\ \left\| \mathbb {M}_{4}\left( \Theta , t, \mathcal {I}\right) -\mathbb {M}_{4}\left( \Theta , t, \mathcal {I}^{*}\right) \right\| \le \eta _{4}\left\| \mathcal {I}-\mathcal {I}^{*}\right\| , \\ \left\| \mathbb {M}_{5}(\Theta , t, \mathcal {A})-\mathbb {M}_{5}\left( \Theta , t, \mathcal {A}^{*}\right) \right\| \le \eta _{5}\left\| \mathcal {A}-\mathcal {A}^{*}\right\| , \\ \left\| \mathbb {M}_{6}\left( \Theta , t, \mathcal {H} \right) -\mathbb {M}_{6}\left( \Theta , t, \mathcal {H}^{*}\right) \right\| \le \eta _{6}\left\| \mathcal {H}-\mathcal {H}^{*}\right\| , \\ \left\| \mathbb {M}_{7}(\Theta , t, \mathcal {R})-\mathbb {M}_{7}\left( \Theta , t, \mathcal {R}^{*}\right) \right\| \le \eta _{7}\left\| \mathcal {R}-\mathcal {R}^{*}\right\| .\\ \end{array} \end{aligned}$$

Where

$$\begin{aligned} \begin{aligned}{}&\eta _{2}=\left( \beta _v+\mu \right) , \quad \eta _{3}=\left( \sigma +\mu \right) , \quad \eta _{4}=\left( \gamma +\mu +\delta \right) , \quad \eta _{5}=\left( \lambda +\mu \right) ,\\&\eta _{6}=\left( \tau +\mu +\delta \right) , \quad \eta _{7}=\left( \eta +\mu \right) . \end{aligned} \end{aligned}$$

The last equation is the Lipschitzian condition that has held for all the mappings. Going in a repetition mode, the equation in (9) becomes

14 $$\begin{aligned} \begin{aligned} \mathcal {S}_{n}(t)-\mathcal {S}(0)=&\frac{1-\Theta }{B(\Theta )} \mathbb {M}_{1}(\Theta , t, \mathcal {S}_{n-1}(t))+\frac{\Theta }{B(\Theta ) \Gamma (\Theta )} \times \int _{0}^{t}(t-\vartheta )^{\Theta -1} \mathbb {M}_{1}(\Theta , \vartheta , \mathcal {S}_{n-1}(\vartheta )) d \vartheta ,\\ \mathcal {V}_{n}(t)-\mathcal {V}(0)=&\frac{1-\Theta }{B(\Theta )} \mathbb {M}_{2}(\Theta , t, \mathcal {V}_{n-1}(t))+\frac{\Theta }{B(\Theta ) \Gamma (\Theta )} \times \int _{0}^{t}(t-\vartheta )^{\Theta -1} \mathbb {M}_{2}(\Theta , \vartheta , \mathcal {V}_{n-1}(\vartheta )) d \vartheta ,\\ \mathcal {E}_{n}(t)-\mathcal {E}(0)=&\frac{1-\Theta }{B(\Theta )} \mathbb {M}_{3}(\Theta , t, \mathcal {E}_{n-1}(t))+\frac{\Theta }{B(\Theta ) \Gamma (\Theta )} \times \int _{0}^{t}(t-\vartheta )^{\Theta -1} \mathbb {M}_{3}(\Theta , \vartheta , \mathcal {E}_{n-1}(\vartheta )) d \vartheta ,\\ \mathcal {I}_{n}(t)-\mathcal {I}(0)=&\frac{1-\Theta }{B(\Theta )} \mathbb {M}_{4}(\Theta , t, \mathcal {I}_{n-1}(t))+\frac{\Theta }{B(\Theta ) \Gamma (\Theta )} \times \int _{0}^{t}(t-\vartheta )^{\Theta -1} \mathbb {M}_{4}(\Theta , \vartheta , \mathcal {I}_{n-1}(\vartheta )) d \vartheta ,\\ \mathcal {A}_{n}(t)-\mathcal {A}(0)=&\frac{1-\Theta }{B(\Theta )} \mathbb {M}_{5}(\Theta , t, \mathcal {A}_{n-1}(t))+\frac{\Theta }{B(\Theta ) \Gamma (\Theta )} \times \int _{0}^{t}(t-\vartheta )^{\Theta -1} \mathbb {M}_{5}(\Theta , \vartheta , \mathcal {A}_{n-1}(\vartheta )) d \vartheta ,\\ \mathcal {H}_{n}(t)-\mathcal {H}(0)=&\frac{1-\Theta }{B(\Theta )} \mathbb {M}_{6}(\Theta , t, \mathcal {H}_{n-1}(t))+\frac{\Theta }{B(\Theta ) \Gamma (\Theta )} \times \int _{0}^{t}(t-\vartheta )^{\Theta -1} \mathbb {M}_{6}(\Theta , \vartheta , \mathcal {H}_{n-1}(\vartheta )) d \vartheta ,\\ \mathcal {R}_{n}(t)-\mathcal {R}(0)=&\frac{1-\Theta }{B(\Theta )} \mathbb {M}_{7}(\Theta , t, R_{n-1}(t))+\frac{\Theta }{B(\Theta ) \Gamma (\Theta )} \times \int _{0}^{t}(t-\vartheta )^{\Theta -1} \mathbb {M}_{7}(\Theta , \vartheta , \mathcal {R}_{n-1}(\vartheta )) d \vartheta . \end{aligned} \end{aligned}$$

together with $\mathcal {S}(0)=\mathcal {S}^0, \mathcal {V}(0)=\mathcal {V}^0, \mathcal {E}(0)=\mathcal {E}^0, \mathcal {I}(0)=\mathcal {I}^0, \mathcal {A}(0)=\mathcal {A}^0, \mathcal {H}(0)=\mathcal {H}^0$ and $\mathcal {R}(0)=\mathcal {R}^0$. Whenever the repeating terms divination is considered, we get

15 $$\begin{aligned} \begin{aligned} \Pi _{\mathcal {S}, n}&= \mathcal {S}_{n}-\mathcal {S}_{n-1}=\frac{1-\Theta }{B(\Theta )} (\mathbb {M}_{1}(\Theta , t, \mathcal {S}_{n-1})-\mathbb {M}_{1}(\Theta , t, \mathcal {S}_{n-2}))\\&\quad +\frac{\Theta }{B(\Theta ) \Gamma (\Theta )} \int _{0}^{t}(t-\vartheta )^{\Theta -1}\left( \mathbb {M}_{1}\left( \Theta , \vartheta , \mathcal {S}_{n-1}(\vartheta )\right) \right. \left. -\mathbb {M}_{1}\left( \Theta , \vartheta , \mathcal {S}_{n-2}(\vartheta )\right) \right) d \vartheta \\ \Pi _{\mathcal {V}, n}&= \mathcal {V}_{n}-\mathcal {V}_{n-1}=\frac{1-\Theta }{B(\Theta )} (\mathbb {M}_{2}(\Theta , t, \mathcal {V}_{n-1})-\mathbb {M}_{2}(\Theta , t, \mathcal {V}_{n-2}))\\&\quad +\frac{\Theta }{B(\Theta ) \Gamma (\Theta )} \int _{0}^{l}(t-\vartheta )^{\Theta -1}\left( \mathbb {M}_{2}\left( \Theta , \vartheta , \mathcal {V}_{n-1}(\vartheta )\right) \right. \left. -\mathbb {M}_{2}\left( \Theta , \vartheta , \mathcal {V}_{n-2}(\vartheta )\right) \right) d \vartheta \\ \Pi _{\mathcal {E}, n}&= \mathcal {E}_{1 n}-\mathcal {E}_{n-1}=\frac{1-\Theta }{B(\Theta )} (\mathbb {M}_{3}(\Theta , t, \mathcal {E}_{n-1})-\mathbb {M}_{3}(\Theta , t, \mathcal {E}_{n-2}))\\&\quad +\frac{\Theta }{B(\Theta ) \Gamma (\Theta )} \int _{0}^{t}(t-\vartheta )^{\Theta -1}\left( \mathbb {M}_{3}\left( \Theta , \vartheta , \mathcal {E}_{n-1}(\vartheta )\right) \right. \left. -\mathbb {M}_{3}\left( \Theta , \vartheta , \mathcal {E}_{n-2}(\vartheta )\right) \right) d \vartheta \\ \Pi _{\mathcal {I}, n}&= \mathcal {I}_{2 n}-\mathcal {I}_{n-1} =\frac{1-\Theta }{B(\Theta )} (\mathbb {M}_{4}(\Theta , t, \mathcal {I}_{n-1})-\mathbb {M}_{4}(\Theta , t, \mathcal {I}_{n-2}))\\&\quad +\frac{\Theta }{B(\Theta ) \Gamma (\Theta )} \int _{0}^{t}(t-\vartheta )^{\Theta -1}\left( \mathbb {M}_{4}\left( \Theta , \vartheta , \mathcal {I}_{n-1}(\vartheta )\right) \right. \left. -\mathbb {M}_{4}\left( \Theta , \vartheta , \mathcal {I}_{n-2}(\vartheta )\right) \right) d \vartheta \\ \Pi _{\mathcal {A}, n}&= \mathcal {A}_{n}-\mathcal {A}_{n-1}=\frac{1-\Theta }{B(\Theta )} (\mathbb {M}_{5}(\Theta , t, \mathcal {A}_{n-1})-\mathbb {M}_{5}(\Theta , t, \mathcal {A}_{n-2}))\\&\quad +\frac{\Theta }{B(\Theta ) \Gamma (\Theta )} \int _{0}^{t}(t-\vartheta )^{\Theta -1}\left( \mathbb {M}_{5}\left( \Theta , \vartheta , \mathcal {A}_{n-1}(\vartheta )\right) \right. \left. -\mathbb {M}_{5}\left( \Theta , \vartheta , \mathcal {A}_{n-2}(\vartheta )\right) \right) d \vartheta \\ \Pi _{\mathcal {H}, n}&= \mathcal {H}_{1 n}-\mathcal {H}_{n-1} =\frac{1-\Theta }{B(\Theta )} (\mathbb {M}_{6}(\Theta , t, \mathcal {H}_{n-1})-\mathbb {M}_{6}(\Theta , t, \mathcal {H}_{n-2}))\\&\quad +\frac{\Theta }{B(\Theta ) \Gamma (\Theta )} \int _{0}^{t}(t-\vartheta )^{\Theta -1}\left( \mathbb {M}_{6}\left( \Theta , \vartheta , \mathcal {H}_{n-1}(\vartheta )\right) \right. \left. -\mathbb {M}_{6}\left( \Theta , \vartheta , \mathcal {H}_{n-2}(\vartheta )\right) \right) d \vartheta \\ \Pi _{\mathcal {R}, n}&= \mathcal {R}_{n}-\mathcal {R}_{n-1}=\frac{1-\Theta }{B(\Theta )} (\mathbb {M}_{7}(\Theta , t, \mathcal {R}_{n-1})-\mathbb {M}_{7}(\Theta , t, \mathcal {R}_{n-2}))\\&\quad +\frac{\Theta }{B(\Theta ) \Gamma (\Theta )} \int _{0}^{t}(t-\vartheta )^{\Theta -1}\left( \mathbb {M}_{7}\left( \Theta , \vartheta , \mathcal {R}_{n-1}(\vartheta )\right) \right. \left. -\mathbb {M}_{7}\left( \Theta , \vartheta , \mathcal {R}_{n-2}(\vartheta )\right) \right) d \vartheta \\ \end{aligned} \end{aligned}$$

It is important to see that

$$\begin{aligned} \begin{aligned}{}&\mathcal {S}_{n}=\sum _{i=0}^{n} \Pi _{(\mathcal {S}, i)},\quad \mathcal {V}_{n}=\sum _{i=0}^{n} \Pi _{(\mathcal {V}, i)}, \quad \mathcal {E}_{n}=\sum _{i=0}^{n} \Pi _{(\mathcal {E}, i)}, \quad \mathcal {I}_{n}=\sum _{i=0}^{n} \Pi _{(\mathcal {I}, i)}\\&\mathcal {A}_{n}=\sum _{i=0}^{n} \Pi _{(\mathcal {A}, i)},\quad \mathcal {H}_{n}=\sum _{i=0}^{n} \Pi _{(\mathcal {H}, i)}, \quad \mathcal {R}_{n}=\sum _{i=0}^{n} \Pi _{(\mathcal {R}, i)}. \end{aligned} \end{aligned}$$

Furthermore, on implication of (12)-(13) and choosing that

$$\begin{aligned} \begin{aligned}{}&\Pi _{\mathcal {S}, n-1}=\mathcal {S}_{n-1}-\mathcal {S}_{n-2}, \quad \Pi _{\mathcal {V}, n-1}=\mathcal {V}_{n-1}-\mathcal {V}_{n-2},\quad \Pi _{\mathcal {E}, n-1}=\mathcal {E}_{n-1}-\mathcal {E}_{n-2}, \\&\Pi _{\mathcal {I}, n-1}=\mathcal {I}_{n-1}-\mathcal {I}_{n-2},\quad \Pi _{\mathcal {A}, n-1}=\mathcal {A}_{n-1}-\mathcal {A}_{n-2}, \quad \Pi _{\mathcal {H}, n-1}=\mathcal {H}_{n-1}-\mathcal {H}_{n-2},\\&\Pi _{\mathcal {R}, n-1}=\mathcal {R}_{n-1}-\mathcal {R}_{n-2}. \end{aligned} \end{aligned}$$

we reach

16 $$\begin{aligned} \begin{array}{l} \left\| \Pi _{\mathcal {S}, n}(t)\right\| \le \frac{1-\Theta }{B(\Theta )} \eta _{1} \left\| \Pi _{\mathcal {S}, n-1}(t)\right\| \frac{\Theta }{B(\Theta ) \Gamma (\Theta )} \eta _{1} \times \int _{0}^{t}(t-\vartheta )^{\Theta -1}\left\| \Pi _{\mathcal {S}, n-1}(\vartheta )\right\| d \vartheta \\ \\ \left\| \Pi _{\mathcal {V}, n}(t)\right\| \le \frac{1-\Theta }{B(\Theta )} \eta _{2} \left\| \Pi _{\mathcal {V}, n-1}(t)\right\| \frac{\Theta }{B(\Theta ) \Gamma (\Theta )} \eta _{2} \times \int _{0}^{t}(t-\vartheta )^{\Theta -1}\left\| \Pi _{\mathcal {V}, n-1}(\vartheta )\right\| d \vartheta \\ \\ \left\| \Pi _{\mathcal {E}, n}(t)\right\| \le \frac{1-\Theta }{B(\Theta )} \eta _{3} \left\| \Pi _{\mathcal {E}, n-1}(t)\right\| \frac{\Theta }{B(\Theta ) \Gamma (\Theta )} \eta _{3} \times \int _{0}^{t}(t-\vartheta )^{\Theta -1}\left\| \Pi _{\mathcal {E}, n-1}(\vartheta )\right\| d \vartheta \\ \\ \left\| \Pi _{\mathcal {I}, n}(t)\right\| \le \frac{1-\Theta }{B(\Theta )} \eta _{4} \left\| \Pi _{\mathcal {I}, n-1}(t)\right\| \frac{\Theta }{B(\Theta ) \Gamma (\Theta )} \eta _{4} \times \int _{0}^{t}(t-\vartheta )^{\Theta -1}\left\| \Pi _{\mathcal {I}, n-1}(\vartheta )\right\| d \vartheta \\ \\ \left\| \Pi _{\mathcal {A}, n}(t)\right\| \le \frac{1-\Theta }{B(\Theta )} \eta _{5} \left\| \Pi _{\mathcal {A}, n-1}(t)\right\| \frac{\Theta }{B(\Theta ) \Gamma (\Theta )} \eta _{5} \times \int _{0}^{t}(t-\vartheta )^{\Theta -1}\left\| \Pi _{\mathcal {A}, n-1}(\vartheta )\right\| d \vartheta \\ \\ \left\| \Pi _{\mathcal {H}, n}(t)\right\| \le \frac{1-\Theta }{B(\Theta )} \eta _{6} \left\| \Pi _{\mathcal {H}, n-1}(t)\right\| \frac{\Theta }{B(\Theta ) \Gamma (\Theta )} \eta _{6} \times \int _{0}^{t}(t-\vartheta )^{\Theta -1}\left\| \Pi _{\mathcal {H}, n-1}(\vartheta )\right\| d \vartheta \\ \\ \left\| \Pi _{\mathcal {R}, n}(t)\right\| \le \frac{1-\Theta }{B(\Theta )} \eta _{7} \left\| \Pi _{\mathcal {R}, n-1}(t)\right\| \frac{\Theta }{B(\Theta ) \Gamma (\Theta )} \eta _{7} \times \int _{0}^{t}(t-\vartheta )^{\Theta -1}\left\| \Pi _{\mathcal {R}, n-1}(\vartheta )\right\| d \vartheta . \end{array} \end{aligned}$$

### Theorem 4.1

Note that the given conditions holds

17 $$\begin{aligned} \frac{1-\Theta }{B(\Theta )} \eta _{i} + \frac{\Theta }{B(\Theta ) \Gamma (\Theta )} b^{\Theta } \eta _{i}<1, i=1,2, \ldots , 7. \end{aligned}$$

Then, (6) has unique root for $t \in [0,b]$.

### Proof

This is derived that $\mathcal {S}(t), \mathcal {V}(t), \mathcal {E}(t), \mathcal {I}(t), \mathcal {A}(t), \mathcal {H}(t)$ and $\mathcal {R}(t)$ are mapping which have bounds. Next, as one can see from Eqs. (12) and (13), the abbreviation $\mathbb {M}_{1}, \mathbb {M}_{2}, \mathbb {M}_{3}, \mathbb {M}_{4}, \mathbb {M}_{5}, \mathbb {M}_{6}$, and $\mathbb {M}_{7}$ holds for Lipchitzian condition. Hence, applying Eq. (16) along with a repeating hypothesis, we get as

18 $$\begin{aligned} \begin{array}{l} \left\| \Pi _{\mathcal {S}, n}(t)\right\| \le \left\| \mathcal {S}_{0}(t)\right\| \left( \frac{1-\Theta }{B(\Theta )} \eta _{1} + \frac{\Theta b^{\Theta }}{B(\Theta ) \Gamma (\Theta )} \eta _{1}\right) ^{n} \\ \\ \left\| \Pi _{\mathcal {V}, n}(t)\right\| \le \left\| \mathcal {V}_{0}(t)\right\| \left( \frac{1-\Theta }{B(\Theta )} \eta _{2} + \frac{\Theta b^{\Theta }}{B(\Theta ) \Gamma (\Theta )} \eta _{2}\right) ^{n} \\ \\ \left\| \Pi _{\mathcal {E}, n}(t)\right\| \le \left\| \mathcal {E}_{0}(t)\right\| \left( \frac{1-\Theta }{B(\Theta )} \eta _{3} + \frac{\Theta b^{\Theta }}{B(\Theta ) \Gamma (\Theta )} \eta _{3}\right) ^{n} \\ \\ \left\| \Pi _{\mathcal {I}, n}(t)\right\| \le \left\| \mathcal {I}_{0}(t)\right\| \left( \frac{1-\Theta }{B(\Theta )} \eta _{4} + \frac{\Theta b^{\Theta }}{B(\Theta ) \Gamma (\Theta )} \eta _{4}\right) ^{n} \\ \\ \left\| \Pi _{\mathcal {A}, n}(t)\right\| \le \left\| \mathcal {A}_{0}(t)\right\| \left( \frac{1-\Theta }{B(\Theta )} \eta _{5} + \frac{\Theta b^{\Theta }}{B(\Theta ) \Gamma (\Theta )} \eta _{5}\right) ^{n}\\ \\ \left\| \Pi _{\mathcal {H}, n}(t)\right\| \le \left\| \mathcal {H}_{0}(t)\right\| \left( \frac{1-\Theta }{B(\Theta )} \eta _{6} + \frac{\Theta b^{\Theta }}{B(\Theta ) \Gamma (\Theta )} \eta _{6}\right) ^{n} \\ \\ \left\| \Pi _{\mathcal {R}, n}(t)\right\| \le \left\| \mathcal {R}_{0}(t)\right\| \left( \frac{1-\Theta }{B(\Theta )} \eta _{7} + \frac{\Theta b^{\Theta }}{B(\Theta ) \Gamma (\Theta )} \eta _{7}\right) ^{n} \end{array} \end{aligned}$$

Therefore, it implies for $n \rightarrow \infty$, all mapping exists and fulfill

$$\begin{aligned} \begin{aligned}{}&\left\| \Pi _{\mathcal {S}, n}\right\| \rightarrow 0, \quad \left\| \Pi _{\mathcal {V}, n}\right\| \rightarrow 0, \quad \left\| \Pi _{\mathcal {E}, n}\right\| \rightarrow 0, \left\| \Pi _{\mathcal {I}, n}\right\| \rightarrow 0,\\&\left\| \Pi _{\mathcal {A}, n}\right\| \rightarrow 0, \quad \left\| \Pi _{\mathcal {H}, n}\right\| \rightarrow 0, \quad \left\| \Pi _{\mathcal {R}, n}\right\| \rightarrow 0. \end{aligned} \end{aligned}$$

Furthermore, from Eq. (18) and applying the triangle in-equality, for any *k*, we have

19 $$\begin{aligned} \begin{array}{l} \left\| \mathcal {S}_{n+k}-\mathcal {S}_{n}\right\| \le \sum _{j=n+1}^{n+k} Z_{1}^{j}=\frac{Z_{1}^{n+1}-Z_{1}^{n+k+1}}{1-Z_{1}}, \\ \\ \left\| \mathcal {V}_{n+k}-\mathcal {V}_{n}\right\| \le \sum _{j=n+1}^{n+k} Z_{2}^{j}=\frac{Z_{2}^{n+1}-Z_{2}^{n+k+1}}{1-Z_{2}}, \\ \\ \left\| \mathcal {E}_{n+k}-\mathcal {E}_{n}\right\| \le \sum _{j=n+1}^{n+k} Z_{3}^{j}=\frac{Z_{3}^{n+1}-Z_{3}^{n+k+1}}{1-Z_{3}}, \\ \\ \left\| \mathcal {I}_{n+k}-\mathcal {I}_{n}\right\| \le \sum _{j=n+1}^{n+k} Z_{4}^{j}=\frac{Z_{4}^{n+1}-Z_{4}^{n+k+1}}{1-Z_{4}}, \\ \\ \left\| \mathcal {A}_{n+k}-\mathcal {A}_{n}\right\| \le \sum _{i=n+1}^{n+k} Z_{5}^{j}=\frac{Z_{5}^{n+1}-Z_{5}^{n+k+1}}{1-Z_{5}}, \\ \\ \left\| \mathcal {H}_{n+k}-\mathcal {H}_{n}\right\| \le \sum _{j=n+1}^{n+k} Z_{6}^{j}=\frac{Z_{6}^{n+1}-Z_{6}^{n+k+1}}{1-Z_{6}}, \\ \\ \left\| \mathcal {R}_{n+k}-\mathcal {R}_{n}\right\| \le \sum _{i=n+1}^{n+k} Z_{7}^{j}=\frac{Z_{7}^{n+1}-Z_{7}^{n+k+1}}{1-Z_{7}}. \end{array} \end{aligned}$$

with $Z_{i}=\frac{1-\Theta }{B(\Theta )} \eta _{i} + \frac{\Theta }{B(\Theta ) \Gamma (\Theta )} b^{\Theta } \eta _{i}<1$ by supposition. As, $\mathcal {S}_{n}, \mathcal {V}_{n}, \mathcal {E}_{n}, \mathcal {I}_{n}, \mathcal {A}_{n}, \mathcal {H}_{n}$ and $\mathcal {R}_{n}$ may be observed as a Cauchy sequence in banach space *B*(*J*). It implies that all the quantities are uniformly convergent^28^. applying the limiting Theorem in Eq. (15) as $n \rightarrow \infty$ conforms that the limit of such type of sequences have unique root of (6). This shows that the existence of Eq. (6) is unique having the condition (17). $\square$

## Hyers-Ulam stability

### Definition 1

. The AB arbitrary order integration problem as in Eq. (9) is called H-U stable^29^ if their exist fixed $\Delta _i >0, i \in {\textbf {N}}^7$ fulfilling: For all$\gamma _i >0, i \in {\textbf {N}}^7$, for

20 $$\begin{aligned} \begin{aligned} | \mathcal {S}(t)-&\frac{1-\Theta }{B(\Theta )} \mathbb {M}_{1}(\Theta , t, \mathcal {S}(t))+\frac{\Theta }{B(\Theta ) \Gamma (\Theta )} \times \int _{0}^{t}(t-\vartheta )^{\Theta -1} \mathbb {M}_{1}(\Theta , \vartheta , \ S(\vartheta )) d \vartheta | \le \gamma _1,\\ |\mathcal {V}(t)-&\frac{1-\Theta }{B(\Theta )} \mathbb {M}_{2}(\Theta , t, \mathcal {V}(t))+\frac{\Theta }{B(\Theta ) \Gamma (\Theta )} \times \int _{0}^{t}(t-\vartheta )^{\Theta -1} \mathbb {M}_{2}(\Theta , \vartheta , \mathcal {V}(\vartheta )) d \vartheta | \le \gamma _2 ,\\ |\mathcal {E}(t)-&\frac{1-\Theta }{B(\Theta )} \mathbb {M}_{3}(\Theta , t, \mathcal {E}(t))+\frac{\Theta }{B(\Theta ) \Gamma (\Theta )} \times \int _{0}^{t}(t-\vartheta )^{\Theta -1} \mathbb {M}_{3}(\Theta , \vartheta , \mathcal {E}(\vartheta )) d \vartheta | \le \gamma _3 ,\\ |\mathcal {I}(t)-&\frac{1-\Theta }{B(\Theta )} \mathbb {M}_{4}(\Theta , t, \mathcal {I}(t)+\frac{\Theta }{B(\Theta ) \Gamma (\Theta )} \times \int _{0}^{t}(t-\vartheta )^{\Theta -1} \mathbb {M}_{4}(\Theta , \vartheta , \mathcal {I}(\vartheta )) d \vartheta | \le \gamma _4 ,\\ |\mathcal {A}(t)-&\frac{1-\Theta }{B(\Theta )} \mathbb {M}_{5}(\Theta , t, \mathcal {A}(t))+\frac{\Theta }{B(\Theta ) \Gamma (\Theta )} \times \int _{0}^{t}(t-\vartheta )^{\Theta -1} \mathbb {M}_{5}(\Theta , \vartheta , \mathcal {A}(\vartheta )) d \vartheta | \le \gamma _5 ,\\ |\mathcal {H}(t)-&\frac{1-\Theta }{B(\Theta )} \mathbb {M}_{6}(\Theta , t, \mathcal {H}(t))+\frac{\Theta }{B(\Theta ) \Gamma (\Theta )} \times \int _{0}^{t}(t-\vartheta )^{\Theta -1} \mathbb {M}_{6}(\Theta , \vartheta , \mathcal {H}(\vartheta )) d \vartheta | \le \gamma _6 ,\\ |\mathcal {R}(t)-&\frac{1-\Theta }{B(\Theta )} \mathbb {M}_{7}(\Theta , t, \mathcal {R}(t))+\frac{\Theta }{B(\Theta ) \Gamma (\Theta )} \times \int _{0}^{t}(t-\vartheta )^{\Theta -1} \mathbb {M}_{7}(\Theta , \vartheta , \mathcal {R}(\vartheta )) d \vartheta | \le \gamma _7. \end{aligned} \end{aligned}$$

there exist $(\dot{\mathcal {S}}, \dot{\mathcal {V}}, \dot{\mathcal {E}}, \dot{\mathcal {I}}, \dot{\mathcal {A}}, \dot{\mathcal {H}}, \dot{\mathcal {R}})$ which are satisfying

21 $$\begin{aligned} \begin{aligned} \dot{\mathcal {S}}(t)=&\frac{1-\Theta }{B(\Theta )} \mathbb {M}_{1}(\Theta , t, \mathcal {S}(t))+\frac{\Theta }{B(\Theta ) \Gamma (\Theta )} \times \int _{0}^{t}(t-\vartheta )^{\Theta -1} \mathbb {M}_{1}(\Theta , \vartheta , \dot{\mathcal {S}}(\vartheta )) d \vartheta ,\\ \dot{\mathcal {V}}(t)=&\frac{1-\Theta }{B(\Theta )} \mathbb {M}_{2}(\Theta , t, \mathcal {V}(t))+\frac{\Theta }{B(\Theta ) \Gamma (\Theta )} \times \int _{0}^{t}(t-\vartheta )^{\Theta -1} \mathbb {M}_{2}(\Theta , \vartheta , \dot{\mathcal {V}}(\vartheta )) d \vartheta ,\\ \dot{\mathcal {E}}(t)=&\frac{1-\Theta }{B(\Theta )} \mathbb {M}_{3}(\Theta , t, \mathcal {E}(t))+\frac{\Theta }{B(\Theta ) \Gamma (\Theta )} \times \int _{0}^{t}(t-\vartheta )^{\Theta -1} \mathbb {M}_{3}(\Theta , \vartheta , \dot{\mathcal {E}}(\vartheta )) d \vartheta ,\\ \dot{\mathcal {I}}(t)=&\frac{1-\Theta }{B(\Theta )} \mathbb {M}_{4}(\Theta , t, \mathcal {I}(t))+\frac{\Theta }{B(\Theta ) \Gamma (\Theta )} \times \int _{0}^{t}(t-\vartheta )^{\Theta -1} \mathbb {M}_{4}(\Theta , \vartheta , \dot{\mathcal {I}}(\vartheta )) d \vartheta ,\\ \dot{\mathcal {A}}(t)=&\frac{1-\Theta }{B(\Theta )} \mathbb {M}_{5}(\Theta , t, \mathcal {A}(t))+\frac{\Theta }{B(\Theta ) \Gamma (\Theta )} \times \int _{0}^{t}(t-\vartheta )^{\Theta -1} \mathbb {M}_{5}(\Theta , \vartheta , \dot{\mathcal {A}}(\vartheta )) d \vartheta ,\\ \dot{\mathcal {H}}(t)=&\frac{1-\Theta }{B(\Theta )} \mathbb {M}_{6}(\Theta , t, \mathcal {H}(t))+\frac{\Theta }{B(\Theta ) \Gamma (\Theta )} \times \int _{0}^{t}(t-\vartheta )^{\Theta -1} \mathbb {M}_{6}(\Theta , \vartheta , \dot{\mathcal {H}}(\vartheta )) d \vartheta ,\\ \dot{\mathcal {R}}(t)=&\frac{1-\Theta }{B(\Theta )} \mathbb {M}_{7}(\Theta , t, \mathcal {R}(t))+\frac{\Theta }{B(\Theta ) \Gamma (\Theta )} \times \int _{0}^{t}(t-\vartheta )^{\Theta -1} \mathbb {M}_{7}(\Theta , \vartheta , \dot{\mathcal {R}}(\vartheta )) d \vartheta . \end{aligned} \end{aligned}$$

Such that

$$\begin{aligned} \begin{aligned}{}&|\mathcal {S}-\dot{\mathcal {S}}| \le \zeta _{1} \gamma _{1}, \quad |\mathcal {V}-\dot{\mathcal {V}}| \le \zeta _{2} \gamma _{2}, \quad |\mathcal {E}-\dot{\mathcal {E}}| \le \zeta _{3} \gamma _{3}, \quad |\mathcal {I}-\dot{\mathcal {I}}| \le \zeta _{4} \gamma _{4},\\&|\mathcal {A}-\dot{\mathcal {A}}| \le \zeta _{5} \gamma _{5}, \quad |\mathcal {H}-\dot{\mathcal {H}}| \le \zeta _{6} \gamma _{6}, \quad |\mathcal {R}-\dot{\mathcal {R}}| \le \zeta _{7} \gamma _{7}. \end{aligned} \end{aligned}$$

### Theorem 5.1

Under the condition *J*, the considered model of arbitrary order (6) is H-U stable.

### Proof

By Theorem (4.1), the proposed AB fractional problem (6) has unique root $({\mathcal {S}}, {\mathcal {V}}, {\mathcal {E}}, {\mathcal {I}},{\mathcal {A}}, {\mathcal {H}}, {\mathcal {R}})$ fulfilling equations of of model (9). Then as follows

22 $$\begin{aligned} \begin{aligned} \Vert \mathcal {S}-\dot{\mathcal {S}}\Vert&\le \frac{1-\Theta }{B(\Theta )}\left\| \mathbb {M}_{1}(\Theta , t, \mathcal {S})-\mathbb {M}_{1}(\Theta , t, \dot{\mathcal {S}})\right\| \\&\quad +\frac{\Theta }{B(\Theta ) \Gamma (\Theta )} \int _{0}^{t}(t-\vartheta )^{\Theta -1} \left\| \mathbb {M}_{1}(\Theta , t, \mathcal {S})-\mathbb {M}_{1}(\Theta , t, \dot{\mathcal {S}})\right\| d \vartheta \\&\le \left[ \frac{1-\Theta }{B(\Theta )}+\frac{\Theta }{B(\Theta ) \Gamma (\Theta )}\right] \Theta _{1}\Vert \mathcal {S}-\dot{\mathcal {S}}\Vert \end{aligned} \end{aligned}$$

23 $$\begin{aligned} \begin{aligned} \Vert \mathcal {V}-\dot{\mathcal {V}}\Vert&\le \frac{1-\Theta }{B(\Theta )}\left\| \mathbb {M}_{2}(\Theta , t, \mathcal {V})-\mathbb {M}_{2}(\Theta , t, \dot{\mathcal {V}})\right\| \\&\quad +\frac{\Theta }{B(\Theta ) \Gamma (\Theta )} \int _{0}^{t}(t-\vartheta )^{\Theta -1} \left\| \mathbb {M}_{2}(\Theta , t, \mathcal {V})-\mathbb {M}_{2}(\Theta , t, \dot{\mathcal {V}})\right\| d \vartheta \\&\le \left[ \frac{1-\Theta }{B(\Theta )}+\frac{\Theta }{B(\Theta ) \Gamma (\Theta )}\right] \Theta _{2}\Vert \mathcal {V}-\dot{\mathcal {V}}\Vert \end{aligned} \end{aligned}$$

24 $$\begin{aligned} \begin{aligned} \Vert \mathcal {E}-\dot{\mathcal {E}}\Vert&\le \frac{1-\Theta }{B(\Theta )}\left\| \mathbb {M}_{3}(\Theta , t, \mathcal {E})-\mathbb {M}_{3}(\Theta , t, \dot{\mathcal {E}})\right\| \\&\quad +\frac{\Theta }{B(\Theta ) \Gamma (\Theta )} \int _{0}^{t}(t-\vartheta )^{\Theta -1} \left\| \mathbb {M}_{3}(\Theta , t, \mathcal {E})-\mathbb {M}_{3}(\Theta , t, \dot{\mathcal {E}})\right\| d \vartheta \\&\le \left[ \frac{1-\Theta }{B(\Theta )}+\frac{\Theta }{B(\Theta ) \Gamma (\Theta )}\right] \Theta _{3}\Vert \mathcal {E}-\dot{\mathcal {E}}\Vert \end{aligned} \end{aligned}$$

25 $$\begin{aligned} \begin{aligned} \Vert \mathcal {I}-\dot{\mathcal {I}}\Vert&\le \frac{1-\Theta }{B(\Theta )}\left\| \mathbb {M}_{4}(\Theta , t, I)-\mathbb {M}_{4}(\Theta , t, \dot{I})\right\| \\&\quad +\frac{\Theta }{B(\Theta ) \Gamma (\Theta )} \int _{0}^{t}(t-\vartheta )^{\Theta -1} \left\| \mathbb {M}_{4}(\Theta , t, \mathcal {I})-\mathbb {M}_{4}(\Theta , t, \dot{\mathcal {I}})\right\| d \vartheta \\&\le \left[ \frac{1-\Theta }{B(\Theta )}+\frac{\Theta }{B(\Theta ) \Gamma (\Theta )}\right] \Theta _{4}\Vert \mathcal {I}-\dot{\mathcal {I}}\Vert \end{aligned} \end{aligned}$$

26 $$\begin{aligned} \begin{aligned} \Vert \mathcal {A}-\dot{\mathcal {A}}\Vert&\le \frac{1-\Theta }{B(\Theta )}\left\| \mathbb {M}_{5}(\Theta , t, \mathcal {A})-\mathbb {M}_{5}(\Theta , t, \dot{\mathcal {A}})\right\| \\&\quad +\frac{\Theta }{B(\Theta ) \Gamma (\Theta )} \int _{0}^{t}(t-\vartheta )^{\Theta -1} \left\| \mathbb {M}_{5}(\Theta , t, A)-\mathbb {M}_{5}(\Theta , t, \dot{\mathcal {A}})\right\| d \vartheta \\&\le \left[ \frac{1-\Theta }{B(\Theta )}+\frac{\Theta }{B(\Theta ) \Gamma (\Theta )}\right] \Theta _{5}\Vert \mathcal {A}-\dot{\mathcal {A}}\Vert \end{aligned} \end{aligned}$$

27 $$\begin{aligned} \begin{aligned} \Vert \mathcal {H}-\dot{\mathcal {H}}\Vert&\le \frac{1-\Theta }{B(\Theta )}\left\| \mathbb {M}_{6}(\Theta , t, \mathcal {H})-\mathbb {M}_{6}(\Theta , t, \dot{\mathcal {H}})\right\| \\&\quad +\frac{\Theta }{B(\Theta ) \Gamma (\Theta )} \int _{0}^{t}(t-\vartheta )^{\Theta -1} \left\| \mathbb {M}_{6}(\Theta , t, \mathcal {H})-\mathbb {M}_{6}(\Theta , t, \dot{\mathcal {H}})\right\| d \vartheta \\&\le \left[ \frac{1-\Theta }{B(\Theta )}+\frac{\Theta }{B(\Theta ) \Gamma (\Theta )}\right] \Theta _{6}\Vert \mathcal {H}-\dot{\mathcal {H}}\Vert \end{aligned} \end{aligned}$$

28 $$\begin{aligned} \begin{aligned} \Vert \mathcal {R}-\dot{\mathcal {R}}\Vert&\le \frac{1-\Theta }{B(\Theta )}\left\| \mathbb {M}_{7}(\Theta , t, \mathcal {R})-\mathbb {M}_{7}(\Theta , t, \dot{\mathcal {R}})\right\| \\&\quad +\frac{\Theta }{B(\Theta ) \Gamma (\Theta )} \int _{0}^{t}(t-\vartheta )^{\Theta -1} \left\| \mathbb {M}_{7}(\Theta , t, R(t))-\mathbb {M}_{7}(\Theta , t, \dot{\mathcal {R}})\right\| d \vartheta \\&\le \left[ \frac{1-\Theta }{B(\Theta )}+\frac{\Theta }{B(\Theta ) \Gamma (\Theta )}\right] \Theta _{7}\Vert \mathcal {R}-\dot{\mathcal {R}}\Vert \end{aligned} \end{aligned}$$

Taking, $\gamma _i = \Theta _i, \Delta _i = \frac{1-\Theta }{B(\Theta )}+\frac{\Theta }{B(\Theta ) \Gamma (\Theta )},$ this implies

29 $$\begin{aligned} \Vert \mathcal {S}-\dot{\mathcal {S}}\Vert \le \gamma _1 \Delta _1 \end{aligned}$$

Similarly, we have the followings

30 $$\begin{aligned} \left\{ \begin{array}{l} \Vert \mathcal {V}-\dot{\mathcal {V}}\Vert \le \gamma _{2} \Delta _{2} \\ \Vert \mathcal {E}-\dot{\mathcal {E}}\Vert \le \gamma _{3} \Delta _{3} \\ \Vert \mathcal {I}-\dot{\mathcal {I}}\Vert \le \gamma _{4} \Delta _{4} \\ \Vert \mathcal {A}-\dot{\mathcal {A}}\Vert \le \gamma _{5} \Delta _{5} \\ \Vert \mathcal {H}-\dot{\mathcal {H}}\Vert \le \gamma _{6} \Delta _{6} \\ \Vert \mathcal {R}-\dot{\mathcal {R}}\Vert \le \gamma _{7} \Delta _{7}. \end{array}\right. \end{aligned}$$

So the the derivation is achieved. $\square$

## Numerical schemes and graphical results

In this section, we apply the new differential and integral operators to the proposed mathematical model of COVID-19. Here, the the classical differential operator will be replaced by the operator with power-law, exponential decay, and Mittag-Leffler kernels.

### Iterative solution by Newton polynomial

In this section, we presented numerical schemes based on the Newton polynomial^41^ for our model. In^42,43^ Atangana and Seda proposed new COVID-19 models and solved by Newton polynomial. Newton’s interpolation is a classical polynomial interpolation approach and plays a significant role in numerical analysis and image processing. The interpolation function of most classical approaches is unique to the given data. At present, Newton’s polynomial interpolation is at the center of research on polynomial interpolation methods. While it has shown good interpolation performance, The Newton-type polynomial interpolation algorithm has many advantages, such as fast convergence a simple and explicit mathematical representation and ease of computation; easy differentiation, integration, and having derivatives of any order. The value of the Newton-type polynomial interpolant function can be adjusted in the interpolant region by choosing appropriate parameter values; according to the actual geometric design needs, the shape of the interpolation curves or surfaces can be adjusted.

We start with Mittag-Leffler kernel

31 $$\begin{aligned} \left\{ \begin{aligned}{}&^{\mathcal {ABC}} \mathbb {D}_{0, t}^{\Theta }[\mathcal {S}(t)] = (1-p)\Pi +\eta \mathcal {R}^{*}-(\beta _s+\mu +v)\mathcal {S}^{*},\\ {}&^{\mathcal {ABC}} \mathbb {D}_{0, t}^{\Theta }[\mathcal {V}(t)] = p\Pi +v\mathcal {S}^{*}-(\beta _v+\mu )\mathcal {V}^{*},\\ {}&^{\mathcal {ABC}} \mathbb {D}_{0, t}^{\Theta }[\mathcal {E}(t)] = \beta _s\mathcal {S}^{*}+\beta _v\mathcal {V}^{*}-(\sigma +\mu )\mathcal {E}^{*},\\ {}&^{\mathcal {ABC}} \mathbb {D}_{0, t}^{\Theta }[\mathcal {I}(t)] = \sigma \psi \mathcal {E}^{*}+\lambda (1-\Theta )\mathcal {A}^{*}-(\gamma +\mu +\delta )\mathcal {I}^{*},\\ {}&^{\mathcal {ABC}} \mathbb {D}_{0, t}^{\Theta }[\mathcal {A}(t)] = \sigma (1-\psi )\mathcal {E}^{*}-(\lambda +\mu )\mathcal {A}^{*},\\ {}&^{\mathcal {ABC}} \mathbb {D}_{0, t}^{\Theta }[\mathcal {H}(t)] = \gamma (1-\kappa )\mathcal {I}^{*}-(\tau +\mu +\delta )\mathcal {H}^{*},\\ {}&^{\mathcal {ABC}} \mathbb {D}_{0, t}^{\Theta }[\mathcal {R}(t)] = \gamma \kappa \mathcal {I}^{*}+\lambda \Theta \mathcal {A}^{*}+\tau \mathcal {H}^{*}-(\eta +\mu )\mathcal {R}^{*}. \end{aligned}\right. \end{aligned}$$

More simply, we can write as follows;

32 $$\begin{aligned} \left\{ \begin{aligned}{}&^{\mathcal {ABC}} \mathbb {D}_{0, t}^{\Theta }[\mathcal {S}(t)] = \mathcal {S}^{*}(t,\mathcal {S},\mathcal {V},\mathcal {E},\mathcal {I},\mathcal {A},\mathcal {H},\mathcal {R}),\\ {}&^{\mathcal {ABC}} \mathbb {D}_{0, t}^{\Theta }[\mathcal {V}(t)] = \mathcal {V}^{*}(t,\mathcal {S},\mathcal {V},\mathcal {E},\mathcal {I},\mathcal {A},\mathcal {H},\mathcal {R}),\\ {}&^{\mathcal {ABC}} \mathbb {D}_{0, t}^{\Theta }[\mathcal {E}(t)] = \mathcal {E}^{*}(t,\mathcal {S},\mathcal {V},\mathcal {E},\mathcal {I},\mathcal {A},\mathcal {H},\mathcal {R}),\\ {}&^{\mathcal {ABC}} \mathbb {D}_{0, t}^{\Theta }[\mathcal {I}(t)] = \mathcal {I}^{*}(t,\mathcal {S},\mathcal {V},\mathcal {E},\mathcal {I},\mathcal {A},\mathcal {H},\mathcal {R}),\\ {}&^{\mathcal {ABC}} \mathbb {D}_{0, t}^{\Theta }[\mathcal {A}(t)] = \mathcal {A}^{*}(t,\mathcal {S},\mathcal {V},\mathcal {E},\mathcal {I},\mathcal {A},\mathcal {H},\mathcal {R}),\\ {}&^{\mathcal {ABC}} \mathbb {D}_{0, t}^{\Theta }[\mathcal {H}(t)] = \mathcal {H}^{*}(t,\mathcal {S},\mathcal {V},\mathcal {E},\mathcal {I},\mathcal {A},\mathcal {H},\mathcal {R}),\\ {}&^{\mathcal {ABC}} \mathbb {D}_{0, t}^{\Theta }[\mathcal {R}(t)] = \mathcal {R}^{*}(t,\mathcal {S},\mathcal {V},\mathcal {E},\mathcal {I},\mathcal {A},\mathcal {H},\mathcal {R}). \end{aligned}\right. \end{aligned}$$

Later on after application of fractional integration with Mittag-Leffler kernel law and plugging Newton polynomial in type of equations, we evaluate our model as follows;

$$\begin{aligned} \mathcal {S}^{a+1}&= \frac{1-\Theta }{AB(\Theta )}+\mathcal {S}^{*}(t_a,\mathcal {S}^a,\mathcal {V}^a,\mathcal {E}^a,\mathcal {I}^a,\mathcal {A}^a,\mathcal {H}^a,\mathcal {R}^a)\\&\quad +\frac{\Theta (\Delta t)^{\Theta }}{AB(\Theta )\Gamma (\Theta +1)}\sum _{\mu =2}^{a} \mathcal {S}^{*}(t_{\mu -2},\mathcal {S}^{\mu -2},\mathcal {V}^{\mu -2},\mathcal {E}^{\mu -2},\mathcal {I}^{\mu -2},\mathcal {A}^{\mu -2},\mathcal {H}^{\mu -2},\mathcal {R}^{\mu -2})\Pi \\&\quad +\frac{\Theta (\Delta t)^{\Theta }}{AB(\Theta )\Gamma (\Theta +2)}\sum _{\mu =2}^{a} \begin{bmatrix} \mathcal {S}^{*}(t_{\mu -1},\mathcal {S}^{\mu -1},\mathcal {V}^{\mu -1},\mathcal {E}^{\mu -1},\mathcal {I}^{\mu -1},\mathcal {A}^{\mu -1},\mathcal {H}^{\mu -1},\mathcal {R}^{\mu -1}) \\ -\mathcal {S}^{*}(t_{\mu -2},\mathcal {S}^{\mu -2},\mathcal {V}^{\mu -2},\mathcal {E}^{\mu -2},\mathcal {I}^{\mu -2},\mathcal {A}^{\mu -2},\mathcal {H}^{\mu -2},\mathcal {R}^{\mu -2}) \end{bmatrix}\Sigma \\&\quad +\frac{\Theta (\Delta t)^{\Theta }}{2AB(\Theta )\Gamma (\Theta +3)}\sum _{\mu =2}^{a} \begin{Bmatrix} \mathcal {S}^{*}(t_{\mu },\mathcal {S}^{\mu },\mathcal {V}^{\mu },\mathcal {E}^{\mu },\mathcal {I}^{\mu },\mathcal {A}^{\mu },\mathcal {H}^{\mu },\mathcal {R}^{\mu }) \\ -2\mathcal {S}^{*}(t_{\mu -1},\mathcal {S}^{\mu -1},\mathcal {V}^{\mu -1},\mathcal {E}^{\mu -1},\mathcal {I}^{\mu -1},\mathcal {A}^{\mu -1},\mathcal {H}^{\mu -1},\mathcal {R}^{\mu -1})\\ +\mathcal {S}^{*}(t_{\mu -2},\mathcal {S}^{\mu -2},\mathcal {V}^{\mu -2},\mathcal {E}^{\mu -2},\mathcal {I}^{\mu -2},\mathcal {A}^{\mu -2},\mathcal {H}^{\mu -2},\mathcal {R}^{\mu -2}) \end{Bmatrix}\Delta \end{aligned}$$

$$ \begin{aligned} \mathcal {V}^{a+1}&= \frac{1-\Theta }{AB(\Theta )}+\mathcal {V}^{*}(t_a,\mathcal {S}^a,\mathcal {V}^a,\mathcal {E}^a,\mathcal {I}^a,\mathcal {A}^a,\mathcal {H}^a,\mathcal {R}^a)\\&\quad +\frac{\Theta (\Delta t)^{\Theta }}{AB(\Theta )\Gamma (\Theta +1)}\sum _{\mu =2}^{a} \mathcal {V}^{*}(t_{\mu -2},\mathcal {S}^{\mu -2},\mathcal {V}^{\mu -2},\mathcal {E}^{\mu -2},\mathcal {I}^{\mu -2},\mathcal {A}^{\mu -2},\mathcal {H}^{\mu -2},\mathcal {R}^{\mu -2})\Pi \\&\quad +\frac{\Theta (\Delta t)^{\Theta }}{AB(\Theta )\Gamma (\Theta +2)}\sum _{\mu =2}^{a} \begin{bmatrix} \mathcal {V}^{*}(t_{\mu -1},\mathcal {S}^{\mu -1},\mathcal {V}^{\mu -1},\mathcal {E}^{\mu -1},\mathcal {I}^{\mu -1},\mathcal {A}^{\mu -1},\mathcal {H}^{\mu -1},\mathcal {R}^{\mu -1}) \\ -\mathcal {V}^{*}(t_{\mu -2},\mathcal {S}^{\mu -2},\mathcal {V}^{\mu -2},\mathcal {E}^{\mu -2},\mathcal {I}^{\mu -2},\mathcal {A}^{\mu -2},\mathcal {H}^{\mu -2},\mathcal {R}^{\mu -2}) \end{bmatrix}\Sigma \\&\quad +\frac{\Theta (\Delta t)^{\Theta }}{2AB(\Theta )\Gamma (\Theta +3)}\sum _{\mu =2}^{a} \begin{Bmatrix} \mathcal {V}^{*}(t_{\mu },\mathcal {S}^{\mu },\mathcal {V}^{\mu },\mathcal {E}^{\mu },\mathcal {I}^{\mu },\mathcal {A}^{\mu },\mathcal {H}^{\mu },\mathcal {R}^{\mu }) \\ -2\mathcal {V}^{*}(t_{\mu -1},\mathcal {S}^{\mu -1},\mathcal {V}^{\mu -1},\mathcal {E}^{\mu -1},\mathcal {I}^{\mu -1},\mathcal {A}^{\mu -1},\mathcal {H}^{\mu -1},\mathcal {R}^{\mu -1})\\ +\mathcal {V}^{*}(t_{\mu -2},\mathcal {S}^{\mu -2},\mathcal {V}^{\mu -2},\mathcal {E}^{\mu -2},\mathcal {I}^{\mu -2},\mathcal {A}^{\mu -2},\mathcal {H}^{\mu -2},\mathcal {R}^{\mu -2}) \end{Bmatrix}\Delta \end{aligned}$$

$$ \begin{aligned} \mathcal {E}^{a+1}&= \frac{1-\Theta }{AB(\Theta )}+\mathcal {E}^{*}(t_a,\mathcal {S}^a,\mathcal {V}^a,\mathcal {E}^a,\mathcal {I}^a,\mathcal {A}^a,\mathcal {H}^a,\mathcal {R}^a)\\&\quad +\frac{\Theta (\Delta t)^{\Theta }}{AB(\Theta )\Gamma (\Theta +1)}\sum _{\mu =2}^{a} \mathcal {E}^{*}(t_{\mu -2},\mathcal {S}^{\mu -2},\mathcal {V}^{\mu -2},\mathcal {E}^{\mu -2},\mathcal {I}^{\mu -2},\mathcal {A}^{\mu -2},\mathcal {H}^{\mu -2},\mathcal {R}^{\mu -2})\Pi \\&\quad +\frac{\Theta (\Delta t)^{\Theta }}{AB(\Theta )\Gamma (\Theta +2)}\sum _{\mu =2}^{a} \begin{bmatrix} \mathcal {E}^{*}(t_{\mu -1},\mathcal {S}^{\mu -1},\mathcal {V}^{\mu -1},\mathcal {E}^{\mu -1},\mathcal {I}^{\mu -1},\mathcal {A}^{\mu -1},\mathcal {H}^{\mu -1},\mathcal {R}^{\mu -1}) \\ -\mathcal {E}^{*}(t_{\mu -2},\mathcal {S}^{\mu -2},\mathcal {V}^{\mu -2},\mathcal {E}^{\mu -2},\mathcal {I}^{\mu -2},\mathcal {A}^{\mu -2},\mathcal {H}^{\mu -2},\mathcal {R}^{\mu -2}) \end{bmatrix}\Sigma \\&\quad +\frac{\Theta (\Delta t)^{\Theta }}{2AB(\Theta )\Gamma (\Theta +3)}\sum _{\mu =2}^{a} \begin{Bmatrix} \mathcal {E}^{*}(t_{\mu },\mathcal {S}^{\mu },\mathcal {V}^{\mu },\mathcal {E}^{\mu },\mathcal {I}^{\mu },\mathcal {A}^{\mu },\mathcal {H}^{\mu },\mathcal {R}^{\mu }) \\ -2\mathcal {E}^{*}(t_{\mu -1},\mathcal {S}^{\mu -1},\mathcal {V}^{\mu -1},\mathcal {E}^{\mu -1},\mathcal {I}^{\mu -1},\mathcal {A}^{\mu -1},\mathcal {H}^{\mu -1},\mathcal {R}^{\mu -1})\\ +\mathcal {E}^{*}(t_{\mu -2},\mathcal {S}^{\mu -2},\mathcal {V}^{\mu -2},\mathcal {E}^{\mu -2},\mathcal {I}^{\mu -2},\mathcal {A}^{\mu -2},\mathcal {H}^{\mu -2},\mathcal {R}^{\mu -2}) \end{Bmatrix}\Delta \end{aligned}$$

$$ \begin{aligned} \mathcal {I}^{a+1}&= \frac{1-\Theta }{AB(\Theta )}+\mathcal {I}^{*}(t_a,\mathcal {S}^a,\mathcal {V}^a,\mathcal {E}^a,\mathcal {I}^a,\mathcal {A}^a,\mathcal {H}^a,\mathcal {R}^a)\\&\quad +\frac{\Theta (\Delta t)^{\Theta }}{AB(\Theta )\Gamma (\Theta +1)}\sum _{\mu =2}^{a} \mathcal {I}^{*}(t_{\mu -2},\mathcal {S}^{\mu -2},\mathcal {V}^{\mu -2},\mathcal {E}^{\mu -2},\mathcal {I}^{\mu -2},\mathcal {A}^{\mu -2},\mathcal {H}^{\mu -2},\mathcal {R}^{\mu -2})\Pi \\&\quad +\frac{\Theta (\Delta t)^{\Theta }}{AB(\Theta )\Gamma (\Theta +2)}\sum _{\mu =2}^{a} \begin{bmatrix} \mathcal {I}^{*}(t_{\mu -1},\mathcal {S}^{\mu -1},\mathcal {V}^{\mu -1},\mathcal {E}^{\mu -1},\mathcal {I}^{\mu -1},\mathcal {A}^{\mu -1},\mathcal {H}^{\mu -1},\mathcal {R}^{\mu -1}) \\ -\mathcal {I}^{*}(t_{\mu -2},\mathcal {S}^{\mu -2},\mathcal {V}^{\mu -2},\mathcal {E}^{\mu -2},\mathcal {I}^{\mu -2},\mathcal {A}^{\mu -2},\mathcal {H}^{\mu -2},\mathcal {R}^{\mu -2}) \end{bmatrix}\Sigma \\&\quad +\frac{\Theta (\Delta t)^{\Theta }}{2AB(\Theta )\Gamma (\Theta +3)}\sum _{\mu =2}^{a} \begin{Bmatrix} \mathcal {I}^{*}(t_{\mu },\mathcal {S}^{\mu },\mathcal {V}^{\mu },\mathcal {E}^{\mu },\mathcal {I}^{\mu },\mathcal {A}^{\mu },\mathcal {H}^{\mu },\mathcal {R}^{\mu }) \\ -2\mathcal {I}^{*}(t_{\mu -1},\mathcal {S}^{\mu -1},\mathcal {V}^{\mu -1},\mathcal {E}^{\mu -1},\mathcal {I}^{\mu -1},\mathcal {A}^{\mu -1},\mathcal {H}^{\mu -1},\mathcal {R}^{\mu -1})\\ +\mathcal {I}^{*}(t_{\mu -2},\mathcal {S}^{\mu -2},\mathcal {V}^{\mu -2},\mathcal {E}^{\mu -2},\mathcal {I}^{\mu -2},\mathcal {A}^{\mu -2},\mathcal {H}^{\mu -2},\mathcal {R}^{\mu -2}) \end{Bmatrix}\Delta \end{aligned}$$

$$ \begin{aligned} \mathcal {A}^{a+1}&= \frac{1-\Theta }{AB(\Theta )}+\mathcal {A}^{*}(t_a,\mathcal {S}^a,\mathcal {V}^a,\mathcal {E}^a,\mathcal {I}^a,\mathcal {A}^a,\mathcal {H}^a,\mathcal {R}^a)\\&\quad +\frac{\Theta (\Delta t)^{\Theta }}{AB(\Theta )\Gamma (\Theta +1)}\sum _{\mu =2}^{a} \mathcal {A}^{*}(t_{\mu -2},\mathcal {S}^{\mu -2},\mathcal {V}^{\mu -2},\mathcal {E}^{\mu -2},\mathcal {I}^{\mu -2},\mathcal {A}^{\mu -2},\mathcal {H}^{\mu -2},\mathcal {R}^{\mu -2})\Pi \\&\quad +\frac{\Theta (\Delta t)^{\Theta }}{AB(\Theta )\Gamma (\Theta +2)}\sum _{\mu =2}^{a} \begin{bmatrix} \mathcal {A}^{*}(t_{\mu -1},\mathcal {S}^{\mu -1},\mathcal {V}^{\mu -1},\mathcal {E}^{\mu -1},\mathcal {I}^{\mu -1},\mathcal {A}^{\mu -1},\mathcal {H}^{\mu -1},\mathcal {R}^{\mu -1}) \\ -\mathcal {A}^{*}(t_{\mu -2},\mathcal {S}^{\mu -2},\mathcal {V}^{\mu -2},\mathcal {E}^{\mu -2},\mathcal {I}^{\mu -2},\mathcal {A}^{\mu -2},\mathcal {H}^{\mu -2},\mathcal {R}^{\mu -2}) \end{bmatrix}\Sigma \\&\quad +\frac{\Theta (\Delta t)^{\Theta }}{2AB(\Theta )\Gamma (\Theta +3)}\sum _{\mu =2}^{a} \begin{Bmatrix} \mathcal {A}^{*}(t_{\mu },\mathcal {S}^{\mu },\mathcal {V}^{\mu },\mathcal {E}^{\mu },\mathcal {I}^{\mu },\mathcal {A}^{\mu },\mathcal {H}^{\mu },\mathcal {R}^{\mu }) \\ -2\mathcal {A}^{*}(t_{\mu -1},\mathcal {S}^{\mu -1},\mathcal {V}^{\mu -1},\mathcal {E}^{\mu -1},\mathcal {I}^{\mu -1},\mathcal {A}^{\mu -1},\mathcal {H}^{\mu -1},\mathcal {R}^{\mu -1})\\ +\mathcal {A}^{*}(t_{\mu -2},\mathcal {S}^{\mu -2},\mathcal {V}^{\mu -2},\mathcal {E}^{\mu -2},\mathcal {I}^{\mu -2},\mathcal {A}^{\mu -2},\mathcal {H}^{\mu -2},\mathcal {R}^{\mu -2}) \end{Bmatrix}\Delta \end{aligned}$$

$$ \begin{aligned} \mathcal {H}^{a+1}&= \frac{1-\Theta }{AB(\Theta )}+\mathcal {H}^{*}(t_a,\mathcal {S}^a,\mathcal {V}^a,\mathcal {E}^a,\mathcal {I}^a,\mathcal {A}^a,\mathcal {H}^a,\mathcal {R}^a)\\&\quad +\frac{\Theta (\Delta t)^{\Theta }}{AB(\Theta )\Gamma (\Theta +1)}\sum _{\mu =2}^{a} \mathcal {H}^{*}(t_{\mu -2},\mathcal {S}^{\mu -2},\mathcal {V}^{\mu -2},\mathcal {E}^{\mu -2},\mathcal {I}^{\mu -2},\mathcal {A}^{\mu -2},\mathcal {H}^{\mu -2},\mathcal {R}^{\mu -2})\Pi \\&\quad +\frac{\Theta (\Delta t)^{\Theta }}{AB(\Theta )\Gamma (\Theta +2)}\sum _{\mu =2}^{a} \begin{bmatrix} \mathcal {H}^{*}(t_{\mu -1},\mathcal {S}^{\mu -1},\mathcal {V}^{\mu -1},\mathcal {E}^{\mu -1},\mathcal {I}^{\mu -1},\mathcal {A}^{\mu -1},\mathcal {H}^{\mu -1},\mathcal {R}^{\mu -1}) \\ -\mathcal {H}^{*}(t_{\mu -2},\mathcal {S}^{\mu -2},\mathcal {V}^{\mu -2},\mathcal {E}^{\mu -2},\mathcal {I}^{\mu -2},\mathcal {A}^{\mu -2},\mathcal {H}^{\mu -2},\mathcal {R}^{\mu -2}) \end{bmatrix}\Sigma \\&\quad +\frac{\Theta (\Delta t)^{\Theta }}{2AB(\Theta )\Gamma (\Theta +3)}\sum _{\mu =2}^{a} \begin{Bmatrix} \mathcal {H}^{*}(t_{\mu },\mathcal {S}^{\mu },\mathcal {V}^{\mu },\mathcal {E}^{\mu },\mathcal {I}^{\mu },\mathcal {A}^{\mu },\mathcal {H}^{\mu },\mathcal {R}^{\mu }) \\ -2\mathcal {H}^{*}(t_{\mu -1},\mathcal {S}^{\mu -1},\mathcal {V}^{\mu -1},\mathcal {E}^{\mu -1},\mathcal {I}^{\mu -1},\mathcal {A}^{\mu -1},\mathcal {H}^{\mu -1},\mathcal {R}^{\mu -1})\\ +\mathcal {H}^{*}(t_{\mu -2},\mathcal {S}^{\mu -2},\mathcal {V}^{\mu -2},\mathcal {E}^{\mu -2},\mathcal {I}^{\mu -2},\mathcal {A}^{\mu -2},\mathcal {H}^{\mu -2},\mathcal {R}^{\mu -2}) \end{Bmatrix}\Delta \end{aligned}$$

$$ \begin{aligned} \mathcal {R}^{a+1}&= \frac{1-\Theta }{AB(\Theta )}+\mathcal {R}^{*}(t_a,\mathcal {S}^a,\mathcal {V}^a,\mathcal {E}^a,\mathcal {I}^a,\mathcal {A}^a,\mathcal {H}^a,\mathcal {R}^a)\\&\quad +\frac{\Theta (\Delta t)^{\Theta }}{AB(\Theta )\Gamma (\Theta +1)}\sum _{\mu =2}^{a} \mathcal {R}^{*}(t_{\mu -2},\mathcal {S}^{\mu -2},\mathcal {V}^{\mu -2},\mathcal {E}^{\mu -2},\mathcal {I}^{\mu -2},\mathcal {A}^{\mu -2},\mathcal {H}^{\mu -2},\mathcal {R}^{\mu -2})\Pi \\&\quad +\frac{\Theta (\Delta t)^{\Theta }}{AB(\Theta )\Gamma (\Theta +2)}\sum _{\mu =2}^{a} \begin{bmatrix} \mathcal {R}^{*}(t_{\mu -1},\mathcal {S}^{\mu -1},\mathcal {V}^{\mu -1},\mathcal {E}^{\mu -1},\mathcal {I}^{\mu -1},\mathcal {A}^{\mu -1},\mathcal {H}^{\mu -1},\mathcal {R}^{\mu -1}) \\ -\mathcal {R}^{*}(t_{\mu -2},\mathcal {S}^{\mu -2},\mathcal {V}^{\mu -2},\mathcal {E}^{\mu -2},\mathcal {I}^{\mu -2},\mathcal {A}^{\mu -2},\mathcal {H}^{\mu -2},\mathcal {R}^{\mu -2}) \end{bmatrix}\Sigma \\&\quad +\frac{\Theta (\Delta t)^{\Theta }}{2AB(\Theta )\Gamma (\Theta +3)}\sum _{\mu =2}^{a} \begin{Bmatrix} \mathcal {R}^{*}(t_{\mu },\mathcal {S}^{\mu },\mathcal {V}^{\mu },\mathcal {E}^{\mu },\mathcal {I}^{\mu },\mathcal {A}^{\mu },\mathcal {H}^{\mu },\mathcal {R}^{\mu }) \\ -2\mathcal {R}^{*}(t_{\mu -1},\mathcal {S}^{\mu -1},\mathcal {V}^{\mu -1},\mathcal {E}^{\mu -1},\mathcal {I}^{\mu -1},\mathcal {A}^{\mu -1},\mathcal {H}^{\mu -1},\mathcal {R}^{\mu -1})\\ +\mathcal {R}^{*}(t_{\mu -2},\mathcal {S}^{\mu -2},\mathcal {V}^{\mu -2},\mathcal {E}^{\mu -2},\mathcal {I}^{\mu -2},\mathcal {A}^{\mu -2},\mathcal {H}^{\mu -2},\mathcal {R}^{\mu -2}) \end{Bmatrix}\Delta \end{aligned}$$

Where

$$\begin{aligned} \begin{aligned} \Delta&= \begin{bmatrix} (a-\mu +1)^{\Theta }\begin{bmatrix} 2(a-\mu )^2+(3\Theta +10)(a-\mu ) \\ +2\mathcal {A}^2+9\Theta +12 \end{bmatrix} \\ -(a-\mu )^{\Theta }\begin{bmatrix} 2(a-\mu )^2+(5\Theta +10)(a-\mu ) \\ +6\mathcal {A}^2+18\Theta +12 \end{bmatrix} \end{bmatrix} ,\nonumber \\ \Sigma&= \begin{bmatrix} (a-\mu +1)^{\Theta }(a-\mu +3+2\Theta ) \\ -(a-\mu )^{\Theta }(a-\mu +3+3\Theta ) \end{bmatrix} ,\\ \Pi&= [(a-\mu +1)^{\Theta }-(a-\mu )^{\Theta }]. \end{aligned} \end{aligned}$$

#### Graphical results

This section examines the numerical simulation results for the COVID-19 pandemic disease model (6). The numerical method employed in the system (6) hinged on Newton’s polynomial Rule. The numerical simulation is undertaken to make use of parameter values from Table 1.

**Figure 3:**
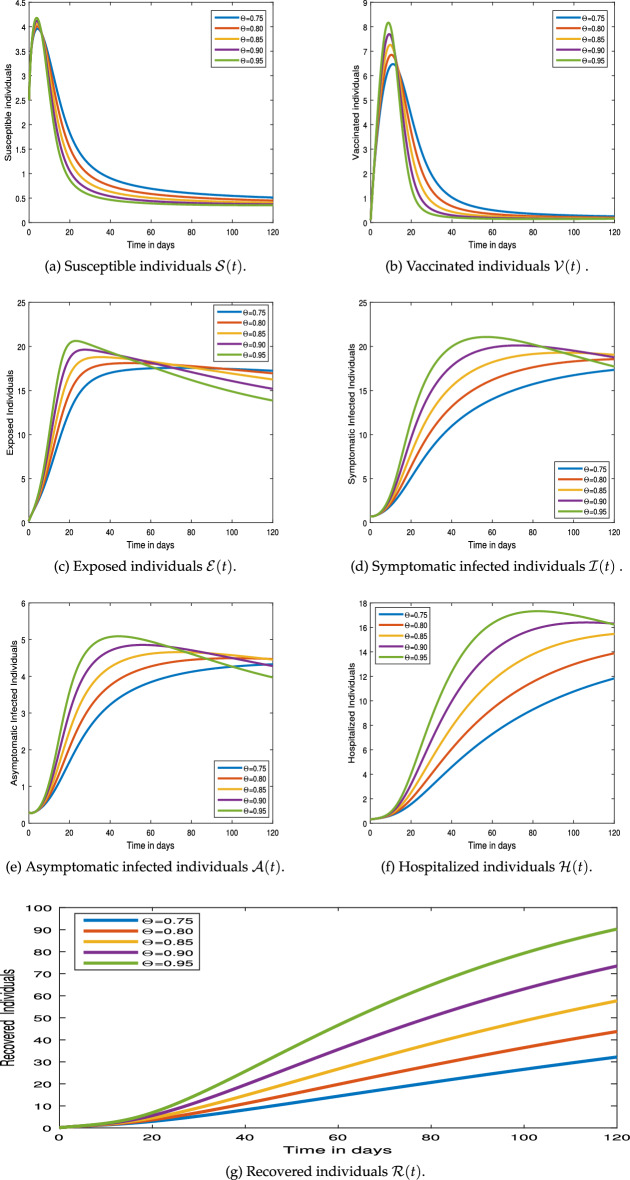
Numerical simulation for COVID-19 epidemic model 6 via Mittag-Leffler Generalized Function.

### Simulation with exponential kernel

Later on by application of fractional integration with exponential kernels and plugging Newton polynomial in such types of equations, we compute our problem as follows;

$$ \begin{aligned} \mathcal {S}^{v+1}&= \mathcal {S}^v+\frac{1-\Theta }{M(\Theta )} \begin{bmatrix} \mathcal {S}^{*}(t_{\mu },\mathcal {S}^{\mu },\mathcal {V}^{\mu },\mathcal {E}^{\mu },\mathcal {I}^{\mu },\mathcal {A}^{\mu },\mathcal {H}^{\mu },\mathcal {R}^{\mu }) \\ -\mathcal {S}^{*}(t_{\mu -1},\mathcal {S}^{\mu -1},\mathcal {V}^{\mu -1},\mathcal {E}^{\mu -1},\mathcal {I}^{\mu -1},\mathcal {A}^{\mu -1},\mathcal {H}^{\mu -1},\mathcal {R}^{\mu -1}) \end{bmatrix}\\&\quad +\frac{\Theta }{M(\Theta )} \begin{Bmatrix} \frac{23}{12}\mathcal {S}^{*}(t_{\mu },\mathcal {S}^{\mu },\mathcal {V}^{\mu },\mathcal {E}^{\mu },\mathcal {I}^{\mu },\mathcal {A}^{\mu },\mathcal {H}^{\mu },\mathcal {R}^{\mu })\Delta t \\ -\frac{4}{3}\mathcal {S}^{*}(t_{\mu -1},\mathcal {S}^{\mu -1},\mathcal {V}^{\mu -1},\mathcal {E}^{\mu -1},\mathcal {I}^{\mu -1},\mathcal {A}^{\mu -1},\mathcal {H}^{\mu -1},\mathcal {R}^{\mu -1})\Delta t\\ +\frac{5}{12}\mathcal {S}^{*}(t_{\mu -2},\mathcal {S}^{\mu -2},\mathcal {V}^{\mu -2},\mathcal {E}^{\mu -2},\mathcal {I}^{\mu -2},\mathcal {A}^{\mu -2},\mathcal {H}^{\mu -2},\mathcal {R}^{\mu -2})\Delta t \end{Bmatrix} \end{aligned}$$

$$\begin{aligned} \mathcal {V}^{v+1}&= \mathcal {V}^v+\frac{1-\Theta }{M(\Theta )} \begin{bmatrix} \mathcal {V}^{*}(t_{\mu },\mathcal {S}^{\mu },\mathcal {V}^{\mu },\mathcal {E}^{\mu },\mathcal {I}^{\mu },\mathcal {A}^{\mu },\mathcal {H}^{\mu },\mathcal {R}^{\mu }) \\ -\mathcal {V}^{*}(t_{\mu -1},\mathcal {S}^{\mu -1},\mathcal {V}^{\mu -1},\mathcal {E}^{\mu -1},\mathcal {I}^{\mu -1},\mathcal {A}^{\mu -1},\mathcal {H}^{\mu -1},\mathcal {R}^{\mu -1}) \end{bmatrix}\\&\quad +\frac{\Theta }{M(\Theta )} \begin{Bmatrix} \frac{23}{12}\mathcal {V}^{*}(t_{\mu },\mathcal {S}^{\mu },\mathcal {V}^{\mu },\mathcal {E}^{\mu },\mathcal {I}^{\mu },\mathcal {A}^{\mu },\mathcal {H}^{\mu },\mathcal {R}^{\mu })\Delta t \\ -\frac{4}{3}\mathcal {V}^{*}(t_{\mu -1},\mathcal {S}^{\mu -1},\mathcal {V}^{\mu -1},\mathcal {E}^{\mu -1},\mathcal {I}^{\mu -1},\mathcal {A}^{\mu -1},\mathcal {H}^{\mu -1},\mathcal {R}^{\mu -1})\Delta t\\ +\frac{5}{12}\mathcal {V}^{*}(t_{\mu -2},\mathcal {S}^{\mu -2},\mathcal {V}^{\mu -2},\mathcal {E}^{\mu -2},\mathcal {I}^{\mu -2},\mathcal {A}^{\mu -2},\mathcal {H}^{\mu -2},\mathcal {R}^{\mu -2})\Delta t \end{Bmatrix} \end{aligned}$$

$$\begin{aligned} \mathcal {E}^{v+1}&= \mathcal {E}^v+\frac{1-\Theta }{M(\Theta )} \begin{bmatrix} \mathcal {E}^{*}(t_{\mu },\mathcal {S}^{\mu },\mathcal {V}^{\mu },\mathcal {E}^{\mu },\mathcal {I}^{\mu },\mathcal {A}^{\mu },\mathcal {H}^{\mu },\mathcal {R}^{\mu }) \\ -\mathcal {E}^{*}(t_{\mu -1},\mathcal {S}^{\mu -1},\mathcal {V}^{\mu -1},\mathcal {E}^{\mu -1},\mathcal {I}^{\mu -1},\mathcal {A}^{\mu -1},\mathcal {H}^{\mu -1},\mathcal {R}^{\mu -1}) \end{bmatrix}\\&\quad +\frac{\Theta }{M(\Theta )} \begin{Bmatrix} \frac{23}{12}\mathcal {E}^{*}(t_{\mu },\mathcal {S}^{\mu },\mathcal {V}^{\mu },\mathcal {E}^{\mu },\mathcal {I}^{\mu },\mathcal {A}^{\mu },\mathcal {H}^{\mu },\mathcal {R}^{\mu })\Delta t \\ -\frac{4}{3}\mathcal {E}^{*}(t_{\mu -1},\mathcal {S}^{\mu -1},\mathcal {V}^{\mu -1},\mathcal {E}^{\mu -1},\mathcal {I}^{\mu -1},\mathcal {A}^{\mu -1},\mathcal {H}^{\mu -1},\mathcal {R}^{\mu -1})\Delta t\\ +\frac{5}{12}\mathcal {E}^{*}(t_{\mu -2},\mathcal {S}^{\mu -2},\mathcal {V}^{\mu -2},\mathcal {E}^{\mu -2},\mathcal {I}^{\mu -2},\mathcal {A}^{\mu -2},\mathcal {H}^{\mu -2},\mathcal {R}^{\mu -2})\Delta t \end{Bmatrix} \end{aligned} $$

$$\begin{aligned} \mathcal {I}^{v+1}&= \mathcal {I}^v+\frac{1-\Theta }{M(\Theta )} \begin{bmatrix} \mathcal {I}^{*}(t_{\mu },\mathcal {S}^{\mu },\mathcal {V}^{\mu },\mathcal {E}^{\mu },\mathcal {I}^{\mu },\mathcal {A}^{\mu },\mathcal {H}^{\mu },\mathcal {R}^{\mu }) \\ -\mathcal {I}^{*}(t_{\mu -1},\mathcal {S}^{\mu -1},\mathcal {V}^{\mu -1},\mathcal {E}^{\mu -1},\mathcal {I}^{\mu -1},\mathcal {A}^{\mu -1},\mathcal {H}^{\mu -1},\mathcal {R}^{\mu -1}) \end{bmatrix}\\&\quad +\frac{\Theta }{M(\Theta )} \begin{Bmatrix} \frac{23}{12}\mathcal {I}^{*}(t_{\mu },\mathcal {S}^{\mu },\mathcal {V}^{\mu },\mathcal {E}^{\mu },\mathcal {I}^{\mu },\mathcal {A}^{\mu },\mathcal {H}^{\mu },\mathcal {R}^{\mu })\Delta t \\ -\frac{4}{3}\mathcal {I}^{*}(t_{\mu -1},\mathcal {S}^{\mu -1},\mathcal {V}^{\mu -1},\mathcal {E}^{\mu -1},\mathcal {I}^{\mu -1},\mathcal {A}^{\mu -1},\mathcal {H}^{\mu -1},\mathcal {R}^{\mu -1})\Delta t\\ +\frac{5}{12}\mathcal {I}^{*}(t_{\mu -2},\mathcal {S}^{\mu -2},\mathcal {V}^{\mu -2},\mathcal {E}^{\mu -2},\mathcal {I}^{\mu -2},\mathcal {A}^{\mu -2},\mathcal {H}^{\mu -2},\mathcal {R}^{\mu -2})\Delta t \end{Bmatrix} \end{aligned} $$

$$\begin{aligned} \mathcal {A}^{v+1}&= \mathcal {A}^v+\frac{1-\Theta }{M(\Theta )} \begin{bmatrix} \mathcal {A}^{*}(t_{\mu },\mathcal {S}^{\mu },\mathcal {V}^{\mu },\mathcal {E}^{\mu },\mathcal {I}^{\mu },\mathcal {A}^{\mu },\mathcal {H}^{\mu },\mathcal {R}^{\mu }) \\ -\mathcal {A}^{*}(t_{\mu -1},\mathcal {S}^{\mu -1},\mathcal {V}^{\mu -1},\mathcal {E}^{\mu -1},\mathcal {I}^{\mu -1},\mathcal {A}^{\mu -1},\mathcal {H}^{\mu -1},\mathcal {R}^{\mu -1}) \end{bmatrix}\\&\quad +\frac{\Theta }{M(\Theta )} \begin{Bmatrix} \frac{23}{12}\mathcal {A}^{*}(t_{\mu },\mathcal {S}^{\mu },\mathcal {V}^{\mu },\mathcal {E}^{\mu },\mathcal {I}^{\mu },\mathcal {A}^{\mu },\mathcal {H}^{\mu },\mathcal {R}^{\mu })\Delta t \\ -\frac{4}{3}\mathcal {A}^{*}(t_{\mu -1},\mathcal {S}^{\mu -1},\mathcal {V}^{\mu -1},\mathcal {E}^{\mu -1},\mathcal {I}^{\mu -1},\mathcal {A}^{\mu -1},\mathcal {H}^{\mu -1},\mathcal {R}^{\mu -1})\Delta t\\ +\frac{5}{12}\mathcal {A}^{*}(t_{\mu -2},\mathcal {S}^{\mu -2},\mathcal {V}^{\mu -2},\mathcal {E}^{\mu -2},\mathcal {I}^{\mu -2},\mathcal {A}^{\mu -2},\mathcal {H}^{\mu -2},\mathcal {R}^{\mu -2})\Delta t \end{Bmatrix} \end{aligned} $$

$$ \begin{aligned} \mathcal {H}^{v+1}&= \mathcal {H}^v+\frac{1-\Theta }{M(\Theta )} \begin{bmatrix} \mathcal {H}^{*}(t_{\mu },\mathcal {S}^{\mu },\mathcal {V}^{\mu },\mathcal {E}^{\mu },\mathcal {I}^{\mu },\mathcal {A}^{\mu },\mathcal {H}^{\mu },\mathcal {R}^{\mu }) \\ -\mathcal {H}^{*}(t_{\mu -1},\mathcal {S}^{\mu -1},\mathcal {V}^{\mu -1},\mathcal {E}^{\mu -1},\mathcal {I}^{\mu -1},\mathcal {A}^{\mu -1},\mathcal {H}^{\mu -1},\mathcal {R}^{\mu -1}) \end{bmatrix}\\&\quad +\frac{\Theta }{M(\Theta )} \begin{Bmatrix} \frac{23}{12}\mathcal {H}^{*}(t_{\mu },\mathcal {S}^{\mu },\mathcal {V}^{\mu },\mathcal {E}^{\mu },\mathcal {I}^{\mu },\mathcal {A}^{\mu },\mathcal {H}^{\mu },\mathcal {R}^{\mu })\Delta t \\ -\frac{4}{3}\mathcal {H}^{*}(t_{\mu -1},\mathcal {S}^{\mu -1},\mathcal {V}^{\mu -1},\mathcal {E}^{\mu -1},\mathcal {I}^{\mu -1},\mathcal {A}^{\mu -1},\mathcal {H}^{\mu -1},\mathcal {R}^{\mu -1})\Delta t\\ +\frac{5}{12}\mathcal {H}^{*}(t_{\mu -2},\mathcal {S}^{\mu -2},\mathcal {V}^{\mu -2},\mathcal {E}^{\mu -2},\mathcal {I}^{\mu -2},\mathcal {A}^{\mu -2},\mathcal {H}^{\mu -2},\mathcal {R}^{\mu -2})\Delta t \end{Bmatrix} \end{aligned}$$

$$ \begin{aligned} \mathcal {R}^{v+1}&= \mathcal {R}^v+\frac{1-\Theta }{M(\Theta )} \begin{bmatrix} \mathcal {R}^{*}(t_{\mu },\mathcal {S}^{\mu },\mathcal {V}^{\mu },\mathcal {E}^{\mu },\mathcal {I}^{\mu },\mathcal {A}^{\mu },\mathcal {H}^{\mu },\mathcal {R}^{\mu }) \\ -\mathcal {R}^{*}(t_{\mu -1},\mathcal {S}^{\mu -1},\mathcal {V}^{\mu -1},\mathcal {E}^{\mu -1},\mathcal {I}^{\mu -1},\mathcal {A}^{\mu -1},\mathcal {H}^{\mu -1},\mathcal {R}^{\mu -1}) \end{bmatrix}\\&\quad +\frac{\Theta }{M(\Theta )} \begin{Bmatrix} \frac{23}{12}\mathcal {R}^{*}(t_{\mu },\mathcal {S}^{\mu },\mathcal {V}^{\mu },\mathcal {E}^{\mu },\mathcal {I}^{\mu },\mathcal {A}^{\mu },\mathcal {H}^{\mu },\mathcal {R}^{\mu })\Delta t \\ -\frac{4}{3}\mathcal {R}^{*}(t_{\mu -1},\mathcal {S}^{\mu -1},\mathcal {V}^{\mu -1},\mathcal {E}^{\mu -1},\mathcal {I}^{\mu -1},\mathcal {A}^{\mu -1},\mathcal {H}^{\mu -1},\mathcal {R}^{\mu -1})\Delta t\\ +\frac{5}{12}\mathcal {R}^{*}(t_{\mu -2},\mathcal {S}^{\mu -2},\mathcal {V}^{\mu -2},\mathcal {E}^{\mu -2},\mathcal {I}^{\mu -2},\mathcal {A}^{\mu -2},\mathcal {H}^{\mu -2},\mathcal {R}^{\mu -2})\Delta t \end{Bmatrix}  \end{aligned}$$

#### Graphical results

This section examines the numerical simulation results for the COVID-19 pandemic disease model (6). The numerical method employed in the system (6) hinged on Newton’s polynomial Rule. The numerical simulation is undertaken to make use of parameter values from Table 1.

**Figure 4:**
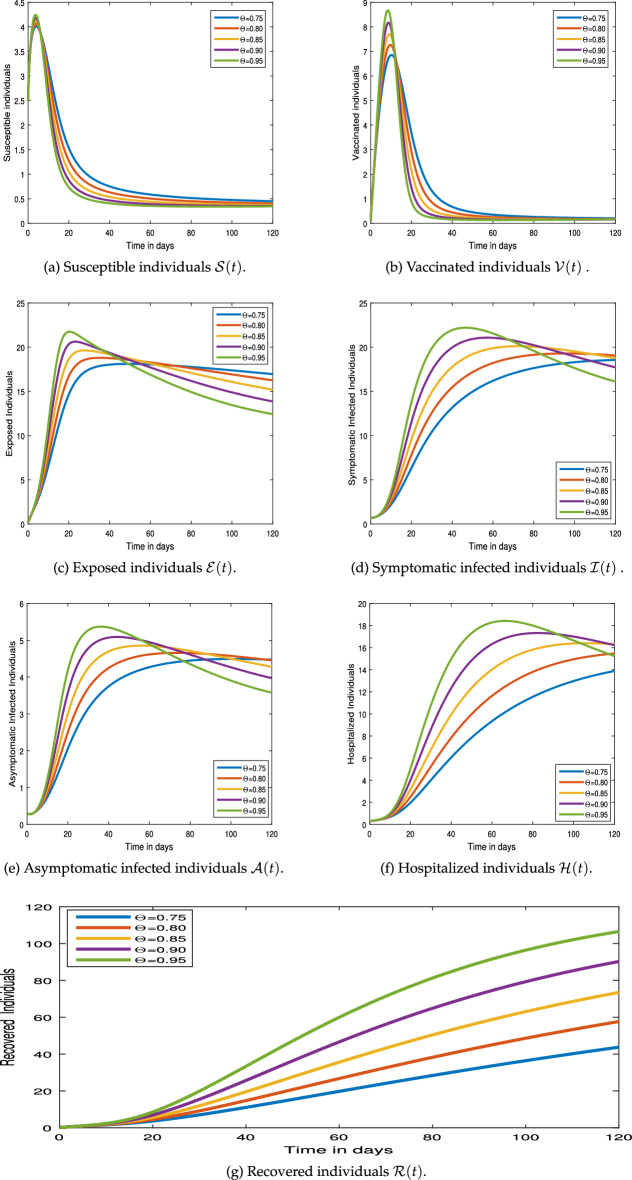
Numerical simulation for COVID-19 epidemic model 6 via exponential kernel.

### Simulation with power law kernel

Finally, we can the following numerical approximation with the Caputo derivative.

$$\begin{aligned}  \mathcal {S}^{a+1}&= \frac{(\Delta t)^{\Theta }}{\Gamma (\Theta +1)}\sum _{\mu =2}^{a} \mathcal {S}^{*}(t_{\mu -2},\mathcal {S}^{\mu -2},\mathcal {V}^{\mu -2},\mathcal {E}^{\mu -2},\mathcal {I}^{\mu -2},\mathcal {A}^{\mu -2},\mathcal {H}^{\mu -2},\mathcal {R}^{\mu -2})\Pi \\&\quad +\frac{(\Delta t)^{\Theta }}{\Gamma (\Theta +2)}\sum _{\mu =2}^{a} \begin{bmatrix} \mathcal {S}^{*}(t_{\mu -1},\mathcal {S}^{\mu -1},\mathcal {V}^{\mu -1},\mathcal {E}^{\mu -1},\mathcal {I}^{\mu -1},\mathcal {A}^{\mu -1},\mathcal {H}^{\mu -1},\mathcal {R}^{\mu -1}) \\ -\mathcal {S}^{*}(t_{\mu -2},\mathcal {S}^{\mu -2},\mathcal {V}^{\mu -2},\mathcal {E}^{\mu -2},\mathcal {I}^{\mu -2},\mathcal {A}^{\mu -2},\mathcal {H}^{\mu -2},\mathcal {R}^{\mu -2}) \end{bmatrix}\Sigma \\&\quad +\frac{(\Delta t)^{\Theta }}{\Gamma (\Theta +3)}\sum _{\mu =2}^{a} \begin{Bmatrix} \mathcal {S}^{*}(t_{\mu },\mathcal {S}^{\mu },\mathcal {V}^{\mu },\mathcal {E}^{\mu },\mathcal {I}^{\mu },\mathcal {A}^{\mu },\mathcal {H}^{\mu },\mathcal {R}^{\mu }) \\ -2\mathcal {S}^{*}(t_{\mu -1},\mathcal {S}^{\mu -1},\mathcal {V}^{\mu -1},\mathcal {E}^{\mu -1},\mathcal {I}^{\mu -1},\mathcal {A}^{\mu -1},\mathcal {H}^{\mu -1},\mathcal {R}^{\mu -1})\\ +\mathcal {S}^{*}(t_{\mu -2},\mathcal {S}^{\mu -2},\mathcal {V}^{\mu -2},\mathcal {E}^{\mu -2},\mathcal {I}^{\mu -2},\mathcal {A}^{\mu -2},\mathcal {H}^{\mu -2},\mathcal {R}^{\mu -2}) \end{Bmatrix}\Delta \end{aligned}$$

$$\begin{aligned} \mathcal {V}^{a+1}&= \frac{(\Delta t)^{\Theta }}{\Gamma (\Theta +1)}\sum _{\mu =2}^{a} \mathcal {V}^{*}(t_{\mu -2},\mathcal {S}^{\mu -2},\mathcal {V}^{\mu -2},\mathcal {E}^{\mu -2},\mathcal {I}^{\mu -2},\mathcal {A}^{\mu -2},\mathcal {H}^{\mu -2},\mathcal {R}^{\mu -2})\Pi \\&\quad +\frac{(\Delta t)^{\Theta }}{\Gamma (\Theta +2)}\sum _{\mu =2}^{a} \begin{bmatrix} \mathcal {V}^{*}(t_{\mu -1},\mathcal {S}^{\mu -1},\mathcal {V}^{\mu -1},\mathcal {E}^{\mu -1},\mathcal {I}^{\mu -1},\mathcal {A}^{\mu -1},\mathcal {H}^{\mu -1},\mathcal {R}^{\mu -1}) \\ -\mathcal {V}^{*}(t_{\mu -2},\mathcal {S}^{\mu -2},\mathcal {V}^{\mu -2},\mathcal {E}^{\mu -2},\mathcal {I}^{\mu -2},\mathcal {A}^{\mu -2},\mathcal {H}^{\mu -2},\mathcal {R}^{\mu -2}) \end{bmatrix}\Sigma \\&\quad +\frac{(\Delta t)^{\Theta }}{\Gamma (\Theta +3)}\sum _{\mu =2}^{a} \begin{Bmatrix} \mathcal {V}^{*}(t_{\mu },\mathcal {S}^{\mu },\mathcal {V}^{\mu },\mathcal {E}^{\mu },\mathcal {I}^{\mu },\mathcal {A}^{\mu },\mathcal {H}^{\mu },\mathcal {R}^{\mu }) \\ -2\mathcal {V}^{*}(t_{\mu -1},\mathcal {S}^{\mu -1},\mathcal {V}^{\mu -1},\mathcal {E}^{\mu -1},\mathcal {I}^{\mu -1},\mathcal {A}^{\mu -1},\mathcal {H}^{\mu -1},\mathcal {R}^{\mu -1})\\ +\mathcal {V}^{*}(t_{\mu -2},\mathcal {S}^{\mu -2},\mathcal {V}^{\mu -2},\mathcal {E}^{\mu -2},\mathcal {I}^{\mu -2},\mathcal {A}^{\mu -2},\mathcal {H}^{\mu -2},\mathcal {R}^{\mu -2}) \end{Bmatrix}\Delta \end{aligned} $$

$$\begin{aligned} \mathcal {E}^{a+1}&=\frac{(\Delta t)^{\Theta }}{\Gamma (\Theta +1)}\sum _{\mu =2}^{a} \mathcal {E}^{*}(t_{\mu -2},\mathcal {S}^{\mu -2},\mathcal {V}^{\mu -2},\mathcal {E}^{\mu -2},\mathcal {I}^{\mu -2},\mathcal {A}^{\mu -2},\mathcal {H}^{\mu -2},\mathcal {R}^{\mu -2})\Pi \\&\quad +\frac{(\Delta t)^{\Theta }}{\Gamma (\Theta +2)}\sum _{\mu =2}^{a} \begin{bmatrix} \mathcal {E}^{*}(t_{\mu -1},\mathcal {S}^{\mu -1},\mathcal {V}^{\mu -1},\mathcal {E}^{\mu -1},\mathcal {I}^{\mu -1},\mathcal {A}^{\mu -1},\mathcal {H}^{\mu -1},\mathcal {R}^{\mu -1}) \\ -\mathcal {E}^{*}(t_{\mu -2},\mathcal {S}^{\mu -2},\mathcal {V}^{\mu -2},\mathcal {E}^{\mu -2},\mathcal {I}^{\mu -2},\mathcal {A}^{\mu -2},\mathcal {H}^{\mu -2},\mathcal {R}^{\mu -2}) \end{bmatrix}\Sigma \\&\quad +\frac{(\Delta t)^{\Theta }}{\Gamma (\Theta +3)}\sum _{\mu =2}^{a} \begin{Bmatrix} \mathcal {E}^{*}(t_{\mu },\mathcal {S}^{\mu },\mathcal {V}^{\mu },\mathcal {E}^{\mu },\mathcal {I}^{\mu },\mathcal {A}^{\mu },\mathcal {H}^{\mu },\mathcal {R}^{\mu }) \\ -2\mathcal {E}^{*}(t_{\mu -1},\mathcal {S}^{\mu -1},\mathcal {V}^{\mu -1},\mathcal {E}^{\mu -1},\mathcal {I}^{\mu -1},\mathcal {A}^{\mu -1},\mathcal {H}^{\mu -1},\mathcal {R}^{\mu -1})\\ +\mathcal {E}^{*}(t_{\mu -2},\mathcal {S}^{\mu -2},\mathcal {V}^{\mu -2},\mathcal {E}^{\mu -2},\mathcal {I}^{\mu -2},\mathcal {A}^{\mu -2},\mathcal {H}^{\mu -2},\mathcal {R}^{\mu -2}) \end{Bmatrix}\Delta \end{aligned}$$

$$ \begin{aligned} \mathcal {I}^{a+1}&= \frac{(\Delta t)^{\Theta }}{\Gamma (\Theta +1)}\sum _{\mu =2}^{a} \mathcal {I}^{*}(t_{\mu -2},\mathcal {S}^{\mu -2},\mathcal {V}^{\mu -2},\mathcal {E}^{\mu -2},\mathcal {I}^{\mu -2},\mathcal {A}^{\mu -2},\mathcal {H}^{\mu -2},\mathcal {R}^{\mu -2})\Pi \\&\quad +\frac{(\Delta t)^{\Theta }}{\Gamma (\Theta +2)}\sum _{\mu =2}^{a} \begin{bmatrix} \mathcal {I}^{*}(t_{\mu -1},\mathcal {S}^{\mu -1},\mathcal {V}^{\mu -1},\mathcal {E}^{\mu -1},\mathcal {I}^{\mu -1},\mathcal {A}^{\mu -1},\mathcal {H}^{\mu -1},\mathcal {R}^{\mu -1}) \\ -\mathcal {I}^{*}(t_{\mu -2},\mathcal {S}^{\mu -2},\mathcal {V}^{\mu -2},\mathcal {E}^{\mu -2},\mathcal {I}^{\mu -2},\mathcal {A}^{\mu -2},\mathcal {H}^{\mu -2},\mathcal {R}^{\mu -2}) \end{bmatrix}\Sigma \\&\quad +\frac{(\Delta t)^{\Theta }}{\Gamma (\Theta +3)}\sum _{\mu =2}^{a} \begin{Bmatrix} \mathcal {I}^{*}(t_{\mu },\mathcal {S}^{\mu },\mathcal {V}^{\mu },\mathcal {E}^{\mu },\mathcal {I}^{\mu },\mathcal {A}^{\mu },\mathcal {H}^{\mu },\mathcal {R}^{\mu }) \\ -2\mathcal {I}^{*}(t_{\mu -1},\mathcal {S}^{\mu -1},\mathcal {V}^{\mu -1},\mathcal {E}^{\mu -1},\mathcal {I}^{\mu -1},\mathcal {A}^{\mu -1},\mathcal {H}^{\mu -1},\mathcal {R}^{\mu -1})\\ +\mathcal {I}^{*}(t_{\mu -2},\mathcal {S}^{\mu -2},\mathcal {V}^{\mu -2},\mathcal {E}^{\mu -2},\mathcal {I}^{\mu -2},\mathcal {A}^{\mu -2},\mathcal {H}^{\mu -2},\mathcal {R}^{\mu -2}) \end{Bmatrix}\Delta \end{aligned}$$

$$\begin{aligned} \mathcal {A}^{a+1}&= \frac{(\Delta t)^{\Theta }}{\Gamma (\Theta +1)}\sum _{\mu =2}^{a} \mathcal {A}^{*}(t_{\mu -2},\mathcal {S}^{\mu -2},\mathcal {V}^{\mu -2},\mathcal {E}^{\mu -2},\mathcal {I}^{\mu -2},\mathcal {A}^{\mu -2},\mathcal {H}^{\mu -2},\mathcal {R}^{\mu -2})\Pi \\&\quad +\frac{(\Delta t)^{\Theta }}{\Gamma (\Theta +2)}\sum _{\mu =2}^{a} \begin{bmatrix} \mathcal {A}^{*}(t_{\mu -1},\mathcal {S}^{\mu -1},\mathcal {V}^{\mu -1},\mathcal {E}^{\mu -1},\mathcal {I}^{\mu -1},\mathcal {A}^{\mu -1},\mathcal {H}^{\mu -1},\mathcal {R}^{\mu -1}) \\ -\mathcal {A}^{*}(t_{\mu -2},\mathcal {S}^{\mu -2},\mathcal {V}^{\mu -2},\mathcal {E}^{\mu -2},\mathcal {I}^{\mu -2},\mathcal {A}^{\mu -2},\mathcal {H}^{\mu -2},\mathcal {R}^{\mu -2}) \end{bmatrix}\Sigma \\&\quad +\frac{(\Delta t)^{\Theta }}{\Gamma (\Theta +3)}\sum _{\mu =2}^{a} \begin{Bmatrix} \mathcal {A}^{*}(t_{\mu },\mathcal {S}^{\mu },\mathcal {V}^{\mu },\mathcal {E}^{\mu },\mathcal {I}^{\mu },\mathcal {A}^{\mu },\mathcal {H}^{\mu },\mathcal {R}^{\mu }) \\ -2\mathcal {A}^{*}(t_{\mu -1},\mathcal {S}^{\mu -1},\mathcal {V}^{\mu -1},\mathcal {E}^{\mu -1},\mathcal {I}^{\mu -1},\mathcal {A}^{\mu -1},\mathcal {H}^{\mu -1},\mathcal {R}^{\mu -1})\\ +\mathcal {A}^{*}(t_{\mu -2},\mathcal {S}^{\mu -2},\mathcal {V}^{\mu -2},\mathcal {E}^{\mu -2},\mathcal {I}^{\mu -2},\mathcal {A}^{\mu -2},\mathcal {H}^{\mu -2},\mathcal {R}^{\mu -2}) \end{Bmatrix}\Delta \end{aligned}$$

$$\begin{aligned} \mathcal {H}^{a+1}&= \frac{(\Delta t)^{\Theta }}{\Gamma (\Theta +1)}\sum _{\mu =2}^{a} \mathcal {H}^{*}(t_{\mu -2},\mathcal {S}^{\mu -2},\mathcal {V}^{\mu -2},\mathcal {E}^{\mu -2},\mathcal {I}^{\mu -2},\mathcal {A}^{\mu -2},\mathcal {H}^{\mu -2},\mathcal {R}^{\mu -2})\Pi \\&\quad +\frac{(\Delta t)^{\Theta }}{\Gamma (\Theta +2)}\sum _{\mu =2}^{a} \begin{bmatrix} \mathcal {H}^{*}(t_{\mu -1},\mathcal {S}^{\mu -1},\mathcal {V}^{\mu -1},\mathcal {E}^{\mu -1},\mathcal {I}^{\mu -1},\mathcal {A}^{\mu -1},\mathcal {H}^{\mu -1},\mathcal {R}^{\mu -1}) \\ -\mathcal {H}^{*}(t_{\mu -2},\mathcal {S}^{\mu -2},\mathcal {V}^{\mu -2},\mathcal {E}^{\mu -2},\mathcal {I}^{\mu -2},\mathcal {A}^{\mu -2},\mathcal {H}^{\mu -2},\mathcal {R}^{\mu -2}) \end{bmatrix}\Sigma \\&\quad +\frac{(\Delta t)^{\Theta }}{\Gamma (\Theta +3)}\sum _{\mu =2}^{a} \begin{Bmatrix} \mathcal {H}^{*}(t_{\mu },\mathcal {S}^{\mu },\mathcal {V}^{\mu },\mathcal {E}^{\mu },\mathcal {I}^{\mu },\mathcal {A}^{\mu },\mathcal {H}^{\mu },\mathcal {R}^{\mu }) \\ -2\mathcal {H}^{*}(t_{\mu -1},\mathcal {S}^{\mu -1},\mathcal {V}^{\mu -1},\mathcal {E}^{\mu -1},\mathcal {I}^{\mu -1},\mathcal {A}^{\mu -1},\mathcal {H}^{\mu -1},\mathcal {R}^{\mu -1})\\ +\mathcal {H}^{*}(t_{\mu -2},\mathcal {S}^{\mu -2},\mathcal {V}^{\mu -2},\mathcal {E}^{\mu -2},\mathcal {I}^{\mu -2},\mathcal {A}^{\mu -2},\mathcal {H}^{\mu -2},\mathcal {R}^{\mu -2}) \end{Bmatrix}\Delta \end{aligned}$$

$$ \begin{aligned} \mathcal {R}^{a+1}&= \frac{(\Delta t)^{\Theta }}{\Gamma (\Theta +1)}\sum _{\mu =2}^{a} \mathcal {R}^{*}(t_{\mu -2},\mathcal {S}^{\mu -2},\mathcal {V}^{\mu -2},\mathcal {E}^{\mu -2},\mathcal {I}^{\mu -2},\mathcal {A}^{\mu -2},\mathcal {H}^{\mu -2},\mathcal {R}^{\mu -2})\Pi \\&\quad +\frac{(\Delta t)^{\Theta }}{\Gamma (\Theta +2)}\sum _{\mu =2}^{a} \begin{bmatrix} \mathcal {R}^{*}(t_{\mu -1},\mathcal {S}^{\mu -1},\mathcal {V}^{\mu -1},\mathcal {E}^{\mu -1},\mathcal {I}^{\mu -1},\mathcal {A}^{\mu -1},\mathcal {H}^{\mu -1},\mathcal {R}^{\mu -1}) \\ -\mathcal {R}^{*}(t_{\mu -2},\mathcal {S}^{\mu -2},\mathcal {V}^{\mu -2},\mathcal {E}^{\mu -2},\mathcal {I}^{\mu -2},\mathcal {A}^{\mu -2},\mathcal {H}^{\mu -2},\mathcal {R}^{\mu -2}) \end{bmatrix}\Sigma \\&\quad +\frac{(\Delta t)^{\Theta }}{\Gamma (\Theta +3)}\sum _{\mu =2}^{a} \begin{Bmatrix} \mathcal {R}^{*}(t_{\mu },\mathcal {S}^{\mu },\mathcal {V}^{\mu },\mathcal {E}^{\mu },\mathcal {I}^{\mu },\mathcal {A}^{\mu },\mathcal {H}^{\mu },\mathcal {R}^{\mu }) \\ -2\mathcal {R}^{*}(t_{\mu -1},\mathcal {S}^{\mu -1},\mathcal {V}^{\mu -1},\mathcal {E}^{\mu -1},\mathcal {I}^{\mu -1},\mathcal {A}^{\mu -1},\mathcal {H}^{\mu -1},\mathcal {R}^{\mu -1})\\ +\mathcal {R}^{*}(t_{\mu -2},\mathcal {S}^{\mu -2},\mathcal {V}^{\mu -2},\mathcal {E}^{\mu -2},\mathcal {I}^{\mu -2},\mathcal {A}^{\mu -2},\mathcal {H}^{\mu -2},\mathcal {R}^{\mu -2}) \end{Bmatrix}\Delta \end{aligned}$$

#### Graphical results

This section examines the numerical simulation results for the COVID-19 pandemic disease model (6). The numerical method employed in the system (6) hinged on Newton’s polynomial Rule. The numerical simulation is undertaken to make use of parameter values from Table 1.

**Figure 5:**
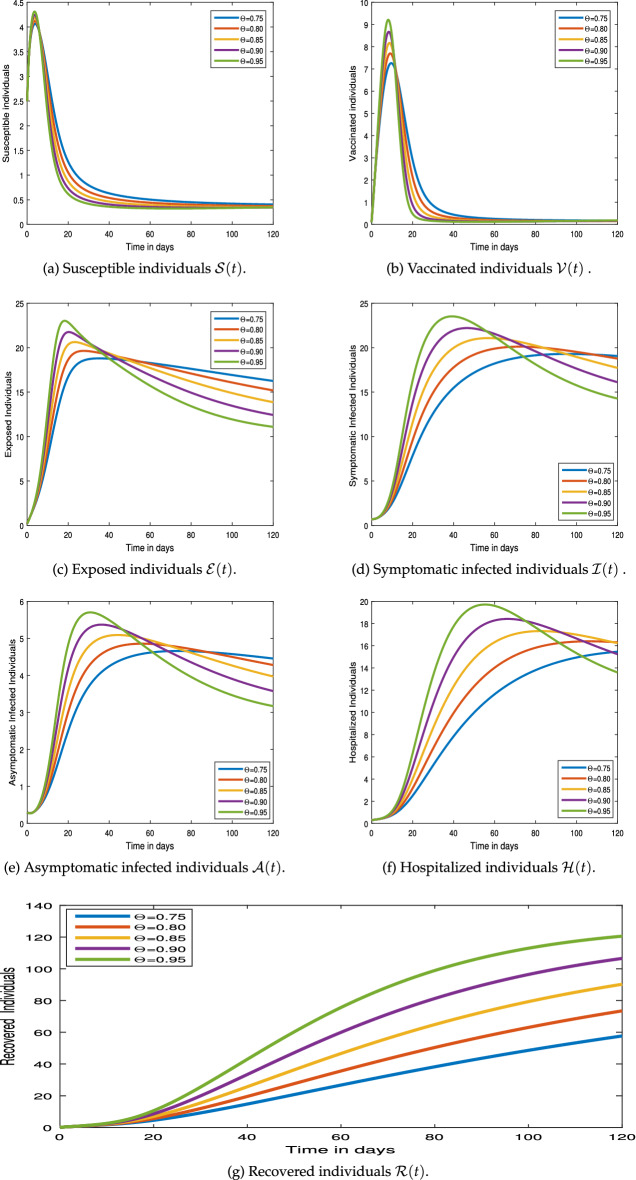
Numerical simulation for COVID-19 epidemic model 6 via power-law kernel

**Figure 6:**
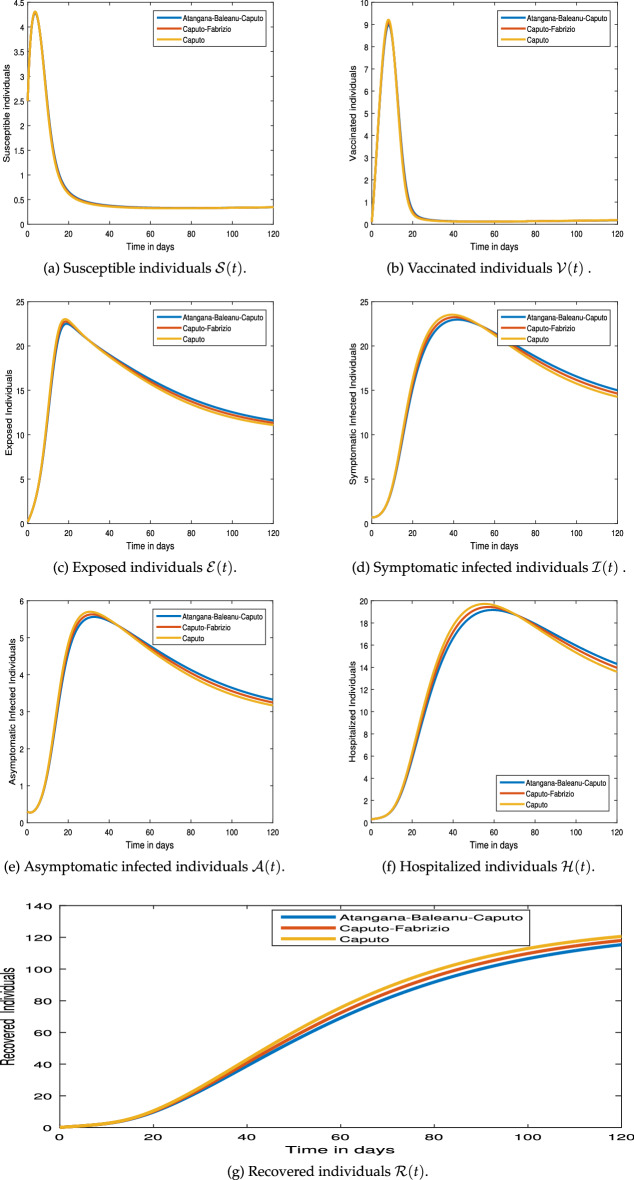
The plot represents the comparison of ABC, CF and Caputo fractional models at $\Theta =0.97$

### Discussion

The key outcomes of the current research work pointed out the infectious system chosen into consideration in the format of arbitrary-order ordinary differential equations in sense of ABC CF and Caputo. For this purpose, we can see clearly Fig. 3a represents the dynamics of $\mathcal {S}(t)$ in various arbitrary orders. We notice that at the beginning there is a rapid increase occurs in the susceptible class in the vicinity of $t= 18$ days. After that phase of time, this agent shows decay and then moves to a stable position. We also see the convergence of the said population to their equilibrium point with the passage of time. Figure 3b represents the vaccinated individuals $\mathcal {V}(t)$ cases which increased slowly for the first some days and then bends for attaining stability. Similarly, Fig. 3c represents the Exposed individuals $\mathcal {E}(t)$ which increased slowly for the first some days and then bends for attaining stability. In the same way, Fig. 3d shows the dynamics of the Symptomatic infected individuals $\mathcal {I}(t)$ against time. We pointed out that the Symptomatic infected individuals show the same dynamics as by $\mathcal {E}(t)$ on various fractional orders. Similarly, Fig. 3e is the increase for the first 40 days and then decrease in the Asymptomatic infected individual’s cases $\mathcal {A}(t)$ on different arbitrary orders of derivatives. One can see that as the order of non-integer order derivative increases, the graph converges to the natural order. In a similar fashion Fig. 3f represents the Hospitalized individuals $\mathcal {H}(t)$ increased rapidly in the vicinity of $t=100$ and then slowly decreased towards the stability point. Figure 3g represents the Recovered cases which increased slowly for the first some days. The said compartment also converges to its equilibrium points with time durations. In the same fashion, Fig. 4 shows all of the seven compartments in different non-integer orders in the CF sense and behaves similar to that of Fig. 3. Also, Fig. 5 represents the simulation results of all of the seven classes in different non-integer orders in the Caputo sense. Similarly, Fig. 6 represents the simulation results of the comparison of the three different operators at $\Theta =0.97$.

## Conclusion

In this work, we address the dynamical behavior of the updated SEIR problem for COVID-19 in sense of Atangana-Baleanu Caputo (ABC) arbitrary order derivative of order $\Theta$ against the required data of Pakistan. The existence of a solution is successfully derived from the concept of the theory of fixed point and functional analysis. For the approximate solution, the arbitrary order polynomial of the Newton method has been applied to the proposed model. The Hyers-Ulam stability is also derived for the proposed model. For numerical simulation, we used the various parameters data of Pakistan and draw each compartment graphically. Finally, given the graphical visualizations to the analytical results to verify the results. We believe that this assumption, extension, and the new analysis are plausible both biologically and mathematically.

## Data Availability

Data sharing is not applicable to this article as no new data were created or analyzed in this study.
